# Cytotoxicity of the methanol extracts and compounds of *Brucea antidysenterica* (Simaroubaceae) towards multifactorial drug-resistant human cancer cell lines

**DOI:** 10.1186/s12906-023-03877-1

**Published:** 2023-02-15

**Authors:** Laetitia M. Youmbi, Yves S. D. Makong, Armelle T. Mbaveng, Simplice B. Tankeo, Ghislain W. Fotso, Bruno L. Ndjakou, Jean D. Wansi, Veronique P. Beng, Norbert Sewald, Bonaventure T. Ngadjui, Thomas Efferth, Victor Kuete

**Affiliations:** 1 Department of Biochemistry, Faculty of Science, University of Dschang, Dschang, Cameroon; 2 Department of Biochemistry, Faculty of Science, University of Yaoundé 1, Yaoundé, Cameroon; 3 Department of Chemistry, Faculty of Science, University of Douala, Douala, Cameroon; 4 Department of Pharmaceutical Biology, Institute of Pharmaceutical and Biomedical Sciences, University of Mainz, Staudinger Weg 5, 55128 Mainz, Germany; 5 Department of Organic Chemistry, Faculty of Science, University of Yaoundé 1, Yaoundé, Cameroon; 6 Department of Chemistry, Higher Teacher Training College, University of Yaoundé 1, Yaounde, Cameroon; 7 Organic and Bioorganic Chemistry, Faculty of Chemistry, Bielefeld University, 33501 Bielefeld, Germany

**Keywords:** Apoptosis, *Brucea antidysenterica*, Cytotoxicity, Multidrug resistance, Hydnocarpin, Simaroubaceae

## Abstract

**Background:**

Cancer remains a global health concern and constitutes an important barrier to increasing life expectancy. Malignant cells rapidly develop drug resistance leading to many clinical therapeutic failures. The importance of medicinal plants as an alternative to classical drug discovery to fight cancer is well known. *Brucea antidysenterica* is an African medicinal plant traditionally used to treat cancer, dysentery, malaria, diarrhea, stomach aches, helminthic infections, fever, and asthma. The present work was designed to identify the cytotoxic constituents of *Brucea antidysenterica* on a broad range of cancer cell lines and to demonstrate the mode of induction of apoptosis of the most active samples.

**Methods:**

Seven phytochemicals were isolated from the leaves (BAL) and stem (BAS) extract of *Brucea antidysenterica* by column chromatography and structurally elucidated using spectroscopic techniques. The antiproliferative effects of the crude extracts and compounds against 9 human cancer cell lines were evaluated by the resazurin reduction assay (RRA). The activity in cell lines was assessed by the Caspase-Glo assay. The cell cycle distribution, apoptosis via propidium iodide (PI) staining, mitochondrial membrane potential (MMP) through 5,5′,6,6′-tetrachloro-1,1′,3,3′-tetraethylbenzimidazolylcarbocyanine iodide (JC-1) staining, and the reactive oxygen species (ROS) via 2´,7´-dichlorodihydrofluoresceine diacetate (H2DCFH-DA) staining, were investigated by flow cytometry.

**Results:**

Phytochemical studies of the botanicals (BAL and BAS) led to the isolation of seven compounds. BAL and its constituents 3, (3-(3-Methyl-1-oxo-2-butenyl))1*H* indole (**1**) and hydnocarpin (**2**), as well as the reference compound, doxorubicin, had antiproliferative activity against 9 cancer cell lines. The IC_50_ values varied from 17.42 µg/mL (against CCRF-CEM leukemia cells) to 38.70 µg/mL (against HCT116 *p53*^*−/−*^ colon adenocarcinoma cells) for BAL, from 19.11 µM (against CCRF-CEM cells) to 47.50 µM (against MDA-MB-231-*BCRP* adenocarcinoma cells) for compound **1**, and from 4.07 µM (against MDA-MB-231-*pcDNA* cells) to 11.44 µM (against HCT116 *p53*^+*/*+^ cells) for compound **2**. Interestingly, hypersensitivity of resistant cancer cells to compound **2** was also observed. BAL and hydnocarpin induced apoptosis in CCRF-CEM cells mediated by caspase activation, the alteration of MMP, and increased ROS levels.

**Conclusion:**

BAL and its constituents, mostly compound **2**, are potential antiproliferative products from *Brucea antidysenterica*. Other studies will be necessary in the perspective of the discovery of new antiproliferative agents to fight against resistance to anticancer drugs.

**Supplementary Information:**

The online version contains supplementary material available at 10.1186/s12906-023-03877-1.

## Background

Cancer remains an incredible human killer and is recognized globally as an important barrier to increasing life expectancy. An incredible number of deaths is linked to tumors in many tissues such as the breast (685 thousand), stomach (769 thousand), liver (830 thousand), colon and rectum (916 thousand), and lung (1,8 million) [1]. It becomes even more dreadful as cancer cells rapidly develop drug-resistant phenotypes, leading to many clinical therapeutic failures and consequently an increased economic burden for patients and societies. Many factors are responsible for the development of recalcitrant tumors. These mostly include the development of resistance to chemically unrelated cytotoxic molecules resulting in the increased energy-dependent proteins that expelled compounds from cells; these also include the insensitivity to apoptosis induced by the cytotoxic molecule as well as the drug-detoxifying mechanisms [2]. Fighting cancer drug resistance appears as a challenging issue in chemotherapy, one of the major modes of treatment [3, 4].

The exploitation of medicinal plants traditionally used under rigorously well-conducted scientific investigations in vitro, in vivo, or in silico, has contributed significantly to the improvement of human health, with many pharmaceuticals deriving from botanicals [5–7].

The search for anticancer drugs capable of counteracting recalcitrant cancer from African medicinal plants has produced interesting results during the past two decades [8–15]. Some African plant extracts that have previously induced hypersensitivity of resistant cancer cells including *Ambrosia maritma* [8], *Imperata cylindrica* [16], *Cola pachycarpa, Curcuma longa* [14]*.* Similarly, some phytochemicals from African medicinal plants possessing corresponding effects include salvimulticanol and salvimulticaoic acid [17], maculine B [18], 5,7-dihydroxy-4'-methoxy-6,8-diprenylisoflavone [19], 8,8-bis-(dihydroconiferyl)-diferulate, aridanin, kihadanin B, progenin III, soyauxinium chloride [20–25].

Throughout Africa, one of the limits of the discovery of antiproliferative drugs remains the absence of clinical studies. Pending the implementation and intensification of clinical studies across the continent, scientists should increase as much as possible the library of bioactive molecules that can later undergo clinical studies. This is the rationale to perform this work aimed at evaluating the cytotoxic potential of botanicals and phytoconstituents of *Brucea antidysenterica* J. F. Mill. (Simaroubaceae) towards refractory cancer cells. The cellular mode of action of leaves methanol extract of *Brucea antidysenterica* (BAL) and its constituent, hydnocarpin (**2**) is also investigated. The plant is an erect tree native to Angola, Cameroon, Congo, Ethiopia, Nigeria, Tanzania, Upper Guinea, and Zimbabwe [26]. *Brucea antidysenterica* is traditionally used to treat cancer, dysentery, malaria, diarrhea, stomach aches, helminthic infections, fever, and asthma [27–29]. Its root and bark extracts earlier displayed the cytotoxic effects against PC-3 (prostate), A-549 (lung), and MCF-7 (breast) cancer cell lines [30]. However, the present study reports for the first time the antiproliferative activities of this plant on various models of drug-resistant cancer cells. Earlier phytochemical investigations of the root and bark of *Brucea antidysenterica* led to the identification of several secondary metabolites belonging to alkaloids, coumarins [30], triterpene quassinoids [29], sterols, fatty acids, and pregnane glycosides [31], and quassinoid glycosides [32] in this plant.

## Methods

### Chemicals

**Fig. 1:**
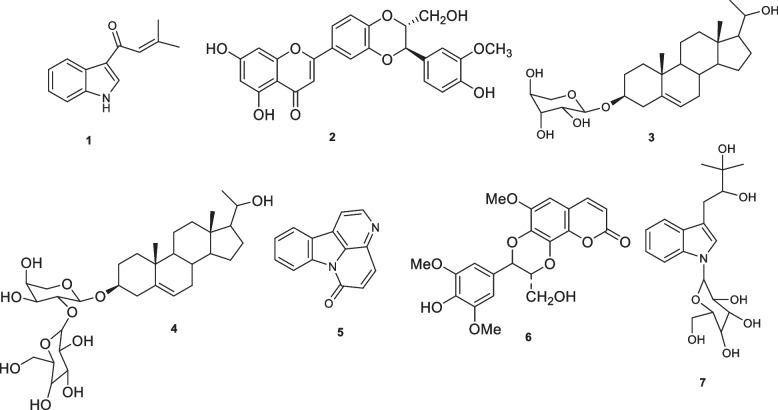
Chemical structure of compounds isolated from the leaves and stem of *Brucea antidysenterica*. 3, (3-(3-Methyl-1-oxo-2-butenyl))1*H* indole (**1**), hydnocarpin (**2**), (20*R*)-*O*-(3)-*α*-_L_-arabinopyranosyl-pregn-5-en-3*β*,20-diol (**3**), (20*R*)-*O*-(3)-*β*-_D_-glucopyranosyl(1 → 2)-*α*-_L_-arabinopyranosyl-pregn -5-en-3*β*,20-diol (**4**), canthin-6-one (**5**), cleomiscosin C (**6**), bruceolline F (**7**)

The phytoconstituents investigated herein were 3, (3-(3-methyl-1-oxo-2-butenyl))1*H* indole (**1**), hydnocarpin (**2**), (20*R*)-*O*-(**3**)-*α*-_L_-arabinopyranosyl-pregn-5-en-3*β*,20-diol (3), (20*R*)-*O*-(3)-*β*-_D_-glucopyranosyl(1 → 2)-*α*-_L_-arabinopyranosyl-pregn -5-en-3*β*,20-diol (**4**), canthin-6-one (**5**), cleomiscosin C (**6**), bruceolline F or 3-(2’,3’-dihydroxy-3’-méthylbutyl)-1N-β-glucopyranosylindole (7) (Fig. 1; Table 1). These compounds were purified from the methanol extracts of the leaves (BAL; **1**–**4**) and stem (BAS; **1**, **5**–**7**) of *Brucea antidysenterica* J. F. Mill. (Simaroubaceae) as described below. Doxorubicin (purity > 98.0%) was as a drug control while geneticin (purity > 98%) was used to maintain the feature of the transfected cells. They were both obtained from Sigma-Aldrich (Taufkirchen Germany). The 5,5′,6,6′-tetrachloro-1,1′,3,3′-tetraethylbenzimidazolylcarbocyanine iodide (JC-1) used for the analysis of mitochondrial membrane potential was purchased from Biomol (Hamburg, Germany). The 2´,7´-dichlorodihydrofluorescein diacetate (H_2_DCFH-DA) used for the evaluation the levels of reactive oxygen species (ROS) as well as the reference molecule for ROS experiment, the hydrogen peroxide (H_2_O_2_), the reference mitochondrial gradient dissipation compound, valinomycin, and dimethyl sulfoxide (DMSO) were also purchased from Sigma-Aldrich.

**Table 1:** General characteristics of the reported phytoconstituents *Brucea antidysenterica*

Compound	Name and formula	Physical aspect	Source	Molecular weight (m/z)	Melting point (°C)	Reference
**1**	3,(3-(3-Methyl-1-oxo-2-butenyl))1H indole (C_13_H_13_NO)	Yellow powder	BAL	199	136–138	[64]
**2**	Hydnocarpin (C_25_H_20_O_9_)	Yellow powder	BAL	464	261–263	[65]
**3**	(20*R*)-*O*-(3)-*α*-_L_-arabinopyranosyl-pregn-5-en-3*β*,20-diol (C_24_H_42_O_6_)	White powder	BAL	473.3	258–260	[66]
**4**	(20*R*)-*O*-(3)-*β*-_D_-glucopyranosyl(1 → 2)-*α*-_L_-arabinopyranosyl-pregn -5-en-3*β*,20-diol (C_32_H_52_O_11_)	White powder	BAL	634	258–260	[67]
**5**	Canthin-6-one (C_14_H_8_N_2_O)	Yellow powder	BAS	220	154–156	[68, 69]
**6**	Cleomiscosin C (C_21_H_20_O_9_)	White powder	BAS	416	254–256	[70]
**7**	Bruceolline F (C_19_H_27_NO_7_)	Yellow powder	BAS	381	209–211	[71]

### Plant material

The stem and leaves of *Brucea antidysenterica* were collected on May 2016 and September 2017 in Bazou in the West region of Cameroon (5° 03′ 60.00" N / 10° 27′ 59.99" E). No authorization to collect the plant sample was needed. The appropriate authorisation has been obtained for the collection of the plant and its use has been carried out in accordance with the relevant guidelines. The plant material (leave, bark, roots, whole plant picture) was authenticated by Mr Nana Victor at the Cameroon National Herbarium in Yaoundé (Voucher specimen number: 54605/HNC).

### Extraction and isolation from leaves

The amounts of 2.2 kg of the air-dried and powdered leaves of *Brucea antidysenterica* were macerated in methanol (MeOH) for 72 h. After evaporation under reduced pressure, the obtained crude or (methanol) extract (60.5 g) was dissolved in hexane/ethyl acetate (AcOEt) 0.5% then 1%. The extract was evaporated to dryness and the residue to give 36 g was submitted to the column chromatography (CC) over silica gel (40–63 μm, 6 × 50 cm) using n-hexane-AcOEt and AcOEt-MeOH gradients as eluents. In total, 321 fractions of 100 ml each were obtained as follows: [(1–15), n-hexane-AcOEt 5%], [(16–32), n-hexane-AcOEt 7.5%], [(33–49), n-hexane-AcOEt 10%], [(50–66), n-hexane-AcOEt 12.5%], [(67–83), n-hexane-AcOEt 15%], [(84–100), n-hexane-AcOEt 17.5%], [(101–117), n-hexane-AcOEt 20%], [(118–134), n-hexane-AcOEt 25%], [(135–151), n-hexane-AcOEt 30%], [(152–168), n-hexane-AcOEt 40%], [(169–185), n-hexane-AcOEt 50%], [(186–202), n-hexane-AcOEt 60%], [(203–219), n-hexane-AcOEt 70%], [(220–236), n-hexane-AcOEt 80%], [(237–253), AcOEt 100%], [(254–270), AcOEt-MeOH 2.5%], [(271–287), AcOEt-MeOH 5%], [(288–304), AcOEt-MeOH 7.5%], [(305–321), AcOEt-MeOH 10%]. Analytic thin layer chromatography (TLC) was used to pooled them into 19 sub-fractions (F_1_′-F_19_′) as follows: fractions 5–11 (F_1’_); 12–29 (F_2’_); 30–45 (F_3’_); 46–60 (F_4’_); 61–81 (F_5’_); 82–97 (F_6’_); 98–116 (F_7’_); 117–133 (F_8’_); 134–150 (F_9’_); 151–164 (F_10’_); 165–185 (F_11’_); 186–202 (F_12’_); 203–220 (F_13’_); 221–234 (F_14’_); 235–250 (F_15’_); 251–269 (F_16’_); 270–282 (F_17’_); 283–305 (F_18’_); 306–321 (F_19’_). Theses fractions afforded compounds** 1** (23.2 mg; yellow powder; from subfraction F_5’_),** 2** (25.9 mg; yellow powder; from subfraction F_10’_),** 3** (13.2 mg; beige powder; from subfraction F_17’_) and** 4** (6.11 mg; white powder; from subfraction F_14’_) after precipitation and filtration.

### Extraction and isolation from the stem

The amounts of 2.3 kg of the air-dried, finely powdered stem of *Brucea antidysenterica* were extracted with MeOH for 72 h. The solution was evaporated in vacuum to yield a brown residue of 24.10 g. The crude extract (22.10 g) was adsorbed on silica (22.10 g) and chromatographed over a silica gel CC (40–63 μm, 4.5 × 50 cm) with n-hexane-ethly acetate (AcOEt) and CHCl_3_-MeOH gradients as eluents. Fractions of 100 ml each were collected as follows: [(1–15), n-hexane 100%], [(16–32), n-hexane-AcOEt 2.5%], [(1–15), n-hexane-AcOEt 5%], [(16–32), n-hexane-AcOEt 7.5%], [(33–49), n-hexane-AcOEt 10%], [(50–66), n-hexane-AcOEt 12.5%], [(67–83), n-hexane-AcOEt 15%], [(84–100), n-hexane-AcOEt 17.5%], [(101–117), n-hexane-AcOEt 20%], [(118–134), n-hexane-AcOEt 25%], [(135–151), n-hexane-AcOEt 30%], [(152–168), n-hexane-AcOEt 40%], [(169–185), n-hexane-AcOEt 50%], [(186–202), n-hexane-AcOEt 60%], [(203–219), n-hexane-AcOEt 70%], [(220–236), n-hexane-AcOEt 80%], [(237–253), AcOEt 100%], [(254–270), AcOEt-MeOH 2.5%], [(271–287), AcOEt-MeOH 5%]. The analytic thin layer chromatography (TLC) was used to pooled them into 19 sub-fractions (F_1_-F_19_*)* as follows: fractions 7–15 (F_1_); 18–29 (F_2_); 32–48 (F_3_); 57–64 (F_4_); 66–81 (F_5_); 83–99 (F_6_); 101–107 (F_7_); 110–129 (F_8_); 133–147 (F_9_); 152–162 (F_10_); 166–184 (F_11_); 187–200 (F_12_); 203–219 (F_13_); 225–230 (F_14_); 234–248 (F_15_); 250–269 (F_16_); 273–289 (F_17_); 291–303 (F_18_); 307–319 (F_19_). Compound **1** (25 mg) was isolated by filtration of the precipitates of the sub-from F_2_. Compounds **5** (13.9 mg) was isolated as beige powder by filtration of the precipitates of the sub-from F_4_. From F_14_, compound **6** (10.5 mg) was obtained respectively as yellow powder. From F_18_, compound **7** (26 mg) was isolated as a white powder.

### Studied cell lines and culture settings

In this work, 9 cancer cell lines with various drug resistance patterns as well as AML12 hepatocytes as normal control cell line were used. The origins of the leukemia cell lines, CCRF-CEM (drug-sensitive) and CEM/ADR5000 (its subline overexpressing multidrug-resistant (MDR) P-glycoprotein (P-gp)) have been reported by several authors [33–35]; Similarly, the source of the HepG2 hepatocarcinoma [36], glioblastoma U87MG cells and its sub-line U87MG.*ΔEGFR* cells transfected with epidermal growth factor receptor (EGFR) [37], HCT116 *p53*^+*/*+^ colon adenocarcinoma cells and its knock-out clone HCT116 *p53*^*−/−*^ cells [38], and MDA-MB-231-*pcDNA3* breast adenocarcinoma cells and breast cancer resistance protein (BCRP)-transfected, and its multidrug-resistant MDA -MB-231-*BCRP* clone 23 cells [39]. The AML12 hepatocytes were used for the comparison of the activities of the samples with HepG2 [40]. CCRF-CEM and CEM/ADR5000 cells were kindly provided by Dr. J. Beck (Department of Pediatrics, University of Greifswald, Greifswald, Germany); MDA-MB-231-pcDNA3 and MDA-MB-231-*BCRP* cells were obtained from Dr. Douglas D. Ross (University of Maryland Greenebaum Cancer Center, University of Maryland School of Medicine, Baltimore, MD); HCT116 *p53*^+*/*+^ and HCT116 *p53*^*−/−*^ cells were a generous gift from Dr. B. Vogelstein and H. Hermeking (Howard Hughes Medical Institute, Baltimore, MD); U87MG and U87MG.ΔEGFR cells were kindly provided by Dr. W. K. Cavenee (Ludwig Institute for Cancer Research, San Diego, CA); HepG2 and AML12 cells were obtained from ATCC (USA). The RPMI-1640 medium was used for the culture of CCRF-CEM and CEM/ADR5000 cells whilst DMEM medium was used for carcinoma cells. The culture media were supplemented (complete medium) with 10% fetal bovine serum and 1% penicillin/streptomycin (Invitrogen, Eggenstein, Germany). The CEM/ADR5000 cell line was maintained in a complete medium containing 5000 ng/ml of doxorubicin to maintain its resistant phenotype. To maintain the resistance phenotype of the resistant carcinoma cell lines such as MDA-MB-231/*BCRP* and HCT116 *p53*^*−/−*^*,* and U87MG.*ΔEGFR* cells, the complete DMEM medium containing geneticin at 800 ng/ml and 400 µg/ml, respectively were used [41, 42].

### Evaluation of the cytotoxicity using resazurin reduction assay (RRA)

The antiproliferative activity of botanicals (BAL and BAS), phytoconstituents (**1–7**) from *Brucea antidysenterica*, and doxorubicin was monitored by RRA [43] under the previously reported work conditions [44–46]. Cells were cultured in a standard condition consisted of a humidified 5% CO_2_ atmosphere and an incubation temperature of 37 °C. The tested concentration ranges were 0.63—80 µg/mL for the botanicals and 0.78—100 µM for phytochemicals. The incubation time of the treated cells was 72 h, and the fluorescence was measured with Infinite M2000 Pro™ plate reader (Tecan, Crailsheim, Germany). The wavelengths used were 544 nm for the excitation and 590 nm for the emission. The IC_50_ values of samples were referred to as the concentrations required to inhibit 50% of the cell proliferation and determined using calibration curve as reported previously [47]. The degree of resistance (D.R.) was set as the ratio of the IC_50_ values of samples in the resistant cell line *vs* that of corresponding sensitive cell line. The selectivity index (S.I.) was the ratio of the IC_50_ value of AML12 hepatocytes *vs.* that of HepG2 cells [13, 48].

### Evaluation of the cell cycle distribution by flow cytometry

The modification of the cell cycle distribution of CCRF-CEM induced by the botanicals BAL, the flavonolignan **2**, the control drug, doxorubicin, or the solvent control DMSO was determined by flow cytometry under the previously reported work conditions [49]. BAL and compound **2** were applied to CCRF-CEM cells (1 ml; 1 × 10^6^ cells) at their ¼ IC_50_, ½ IC_50_, IC_50_, and 2 × IC_50_ values. The whole was then incubated for 24 h as earlier indicated followed by the analysis of the cell cycle distribution with a BD Accury C6 Flow Cytometer (BD Biosciences, Heidelberg, Germany) [49, 50]. Assays were performed three times in triplicates.

### Evaluation of apoptosis by flow cytometry

The annexin V/propidium iodide (PI) staining combined to the flow cytometry was used to detect apoptotic cells in CCRF-CEM cells by BAL, the flavonolignan **2**, doxorubicin, or the solvent (DMSO) [20, 25, 46]. BAL and compound **2**, as well as doxorubicin were applied to CCRF-CEM cells (1 ml; 1 × 10^6^ cells) at their ¼ IC_50_, ½ IC_50_, IC_50_, and 2 × IC_50_ values followed by a 24 h incubation as indicated earlier. Afterward, apoptosis detection was undertaken with a fluorescein isothiocynate (FITC)-conjugated annexin V/PI assay kit (eBioscience™ Annexin V; Invitogen, San Diego, USA). The measurement was done using BD Accury C6 Flow Cytometer (BD Biosciences) under experimental conditions previously reported [20, 25]. The assays were done thrice independently with three repetitions each.

### Evaluation of caspases activity by Caspase-Glo assay

The activity of caspases in CCRF-CEM cells treated with BAL and the flavonolignan **2** was determined by Caspase-Glo assay [42]. BAL and compound **2** were applied to CCRF-CEM cells (100 µl; 1.5 × 10^4^ cells for caspase 3/7 assay or 3 × 10^4^ cells for caspase 8 and caspase 9 assays) at their ½ IC_50_, IC_50_, and 2 × IC_50_. The whole was then incubated for 6 h (standard culture conditions) and the activity of caspases was detected by spectrophotometry as previously described [42].

### Quantification of mitochondrial membrane potential (MMP) alteration and reactive oxygen species (ROS) production by Flow cytometry

The modification of the MMP as well as the ROS levels after the application of BAL, and flavonolignan **2** at ¼ IC_50_, ½ IC_50_, IC_50_, and 2 × IC_50_ to CCRF-CEM cells (1 × 10^6^) was performed by flow cytometry [45]. DMSO was used as solvent control while valinomycin served as a positive control. The cells were incubated for 24 h and further stained for 30 min with JC-1 [45] and measured (1 × 10^4^ cells) in an LSR-Fortessa FACS analyzer (Becton–Dickinson) [45]. For ROS determination, CCEF-CEM cells were similarly treated BAL, the flavonolignan **2**, hydrogen peroxide (H_2_O_2_; positive control), DMSO (solvent control) as in the MMP analysis, then incubated for 24 h. They were further stained with H_2_DCFH-DA and measured (1 × 10^4^ cells) in an LSR-Fortessa FACS analyzer [40, 51, 52].

### Statistical analysis

The Graph Pad Prism 5 software was used for the statistical analysis. Data from independent assays are presented as mean value ± standard deviation. The difference between the mean values of test samples and the control, One-way Analysis Variance (ANOVA) and post hoc Tukey’s test was used to establish the significance. The significant differences were regarded as *p*-value < 0.05.

## Results

### Phytochemistry

Seven phytochemicals were purified from the methanol extracts of the leaves (**1–4**) and stem (**1, 5–7**) of *Brucea antidysenterica*. Their chemical structures (Fig. 1) were determined from their NMR spectra and a comparison with data from the literature. Their general characteristics phytochemicals **1–7** are given in Table 1. They included three alkaloids: 3, (3-(3-Methyl-1-oxo-2-butenyl))1*H* indole (**1**), canthin-6-one (**5**) and bruceolline F (**7**), a flavonolignan, hydnocarpin (**2**), two sterol glycosides: (20*R*)-*O*-(**3**)-*α*-_L_-arabinopyranosyl-pregn-5-en-3*β*,20-diol (3) and (20*R*)-*O*-(3)-*β*-_D_-glucopyranosyl(1 → 2)-*α*-_L_-arabinopyranosyl-pregn -5-en-3*β*,20-diol (**4**), and a coumarinolignans, cleomiscosin C (**6**). The NMR spectra of the isolated phytoconstituents are available as supporting information.

### Cytotoxicity

The cytotoxic effects BAL, BAS, their isolated secondary metabolites as well as doxorubicin were determined by RRA towards the studied cell lines. The results are shown in Tables 2 and 3. The IC_50_ values of BAL (Table 2), phytochemiclas **1** and **2**, and doxorubicin (Table 3) were obtained against the nine cancer cell lines investigated. The other tested samples (BAS, compounds **3–7**) had selective cytotoxic effects. The obtained IC_50_ values varied from 17.42 µg/ml (against CCRF-CEM leukemia cells) to 38.70 µg/mL (against HCT116 *p53*^*−/−*^ colon adenocarcinoma cells) for BAL (Table 2); from 19.11 µM (against CCRF-CEM cells) to 47.50 µM (against MDA-MB-231-*BCRP* breast adenocarcinoma cells) for compound **1**, from 4.07 µM (against MDA-MB-231-*pcDNA* cells) to 11.44 µM (against HCT116 *p53*^+*/*+^ cells) for compound **2**, and from 0.02 µM (against CCRF-CEM cells) to 122.96 µM (against CEM/ADR5000 cells) for doxorubicin. The three most active samples (BAL, compounds **1** and **2**) also displayed lower cytotoxicity (S.I. > 2.12) against AML12 cells *vs* HepG2 cells (Tables 2 and 3). Hypersensitivity (collateral sensitivity) of MDA-MB-231-*BCRP* cells *vs.* MDA-MB-231-*pcDNA* cells (D.R. of 0.51), and U87.MGΔ*EGFR* glioblastoma cells *vs.* U87.MG cells (D.R. of 0.69) to BAL was observed (Table 2). This was also the case for CEM/ADR5000 cells compared to CCRF-CEM cells (D.R. of 0.72), HCT116 *p53*^*−/−*^ cells compared to HCT116 *p53*^+*/*+^ cells (D.R. of 0.59), and U87.MGΔ*EGFR* cells compared to U87.MG cells (D.R. of 0.58) *vis-a-vis* compound **2** (Table 3). Normal sensitivity of CEM/ADR5000 cells compared to CCRF-CEM cells (D.R. of 1.08) and HCT116 *p53*^*−/−*^ cells compared to HCT116 *p53*^+*/*+^ cells (D.R. of 1.04) to BAL was also noted. The tested cancer cells showed cross-resistance to compound **1** (Table 2).

**Table 2:** IC_50_ values of crude extracts from leaves (BAL), stem (BAS) of *Brucea antidysenterica*, and doxorubicin towards the tested cell lines

Cell lines	Samples, IC_50_ ( µg/mL) and D.R.* or S.I.** (in bracket)		
	**Crude extracts**		**Reference drug**
	BAL	**B**AS	**Doxorubicin**
CCRF-CEM	**17.42 ± 0.84**	43.42 ± 2.86	**0.02 ± 0.00**
CEM/ADR5000 Degree of resistance*	**18.84 ± 1.28** (1.08)	**12.43 ± 0.68** (0.29)	122.96 ± 10.94 (6,683.00)
MDA-MB-231-*pcDNA*	36.34 ± 1.99	34.76 ± 1.78	**0.13 ± 0.01**
MDA-MB-231-*BCRP* Degree of resistance	**18.67 ± 1.53** (0.51)	68.12 ± 3.47 (1.96)	**0.79 ± 0.08** (6.14)
HCT116 *p53*^+*/*+^	37.18 ± 2.51	68.91 ± 4.69	**0.48 ± 0.06**
HCT116 *p53*^*−/−*^ Degree of resistance	38.70 ± 3.02 (1.04)	> 80 (> 1.16)	**1.78 ± 0.08** (3.73)
U87MG	36.83 ± 2.55	34.21 ± 1.30	**0.26 ± 0.03**
U87MG.Δ*EGFR* Degree of resistance	25.57 ± 1.84 (0.69)	55.18 ± 3.82 (1.61)	**0.98 ± 0.07** (3.79)
HepG2	36.98 ± 2.41	28.61 ± 0.97	**4.56 ± 0.48**
AML12 Selectivity index**	78.39 ± 4.62 (2.12)	> 80 (> 2.80)	> 50 (> 11.59)

(*): Degree of resistance (D.R.): ratio of IC_50_ *vs* IC_50_ in the sensitive cell line; CEM/ADR5000 *vs* CCRF-CEM, MDA-MB-231-*BCRP vs* MDA-MB-231-*pcDNA,* HCT116 *p53*^*−/−*^* vs* HCT116 *p53*^+*/*+^ and U87.MGΔEGFR *vs* U87.MG; (**): Selectivity index (S.I): ratio of IC_50_ in AML12 *vs* IC_50_ in HepG2 cells [13, 48]; Methanol extract from leaves (BAL), and stem (BAS) of *Brucea antidysenterica*. In bold: significant cytotoxic effect [9, 72]

**Table 3:** IC_50_ values of compounds isolated *Brucea antidysenterica* towards the investigated cell lines

Cell lines	Samples, IC_50_ (in µM) and D.R.* or S.I.** (in bracket)						
	**1**	**2**	**3**	**4**	**5**	**6**	**7**
CCRF-CEM	19.11 ± 1.56	**4.28 ± 0.31**	28.42 ± 1.76	31.99 ± 2.16	44.22 ± 3.52	23.19 ± 1.63	20.38 ± 0.94
CEM/ADR5000 Degree of resistance*	32.03 ± 2.05 (1.68)	**3.07 ± 0.18** (0.72)	73.94 ± 5.32 (2.60)	> 100 (> 3.13)	> 100 (> 2.26)	> 100 (> 4.31)	> 100 (> 4.91)
MDA-MB-231-*pcDNA*	13.40 ± 0.93	**4.07 ± 0.37**	> 100	> 100	> 100	> 100	> 100
MDA-MB-231-*BCRP* Degree of resistance	47.50 ± 2.75 (3.54)	**6.65 ± 0.24** (1.63)	> 100	> 100	> 100	> 100	> 100
HCT116 *p53*^+*/*+^	23.86 ± 2.32	11.44 ± 0.98	> 100	> 100	24.72 ± 2.16	> 100	> 100
HCT116 *p53*^*−/−*^ Degree of resistance	36.43 ± 3.04 (1.53)	**6.74 ± 0.33** (0.59)	50.48 ± 3.81 (< 0.50)	32.16 ± 2.37 (< 0.32)	20.97 ± 1.72 (0.85)	> 100	> 100
U87MG	24.25 ± 1.16	**9.29 ± 0.73**	> 100	28.29 ± 2.08	19.58 ± 1.55	> 100	> 100
U87MG.Δ*EGFR* Degree of resistance	36.28 ± 3.01 (1.50)	**5.42 ± 0.44** (0.58)	> 100	28.32 ± 1.66 (1.00)	21.12 ± 1.49 (1.08)	> 100	> 100
HepG2	39.94 ± 2.84	**2.73 ± 0.19**	85.16 ± 5.70	31.17 ± 2.11	26.09 ± 1.63	15.48 ± 1.06	> 100
AML12 Selectivity index**	> 100 (> 2.50)	51.16 ± 3.20 (18.74)	> 100 (> 1.17)	> 100 (> 3.21)	> 100 (> 3.83)	> 100 (> 6.46)	> 100

(*): (*): Degree of resistance (D.R.): ratio of IC_50_ *vs* IC_50_ in the sensitive cell line; CEM/ADR5000 *vs* CCRF-CEM, MDA-MB-231-*BCRP vs* MDA-MB-231-*pcDNA,* HCT116 *p53*^*−/−*^* vs* HCT116 *p53*^+*/*+^ and U87.MGΔEGFR *vs* U87.MG; (**): Selectivity index (S.I): ratio of IC_50_ in AML12 *vs* IC_50_ in HepG2 cells [13, 48]; Tested samples were: 3, (3-(3-Methyl-1-oxo-2-butenyl))1*H* indole (**1**), hydnocarpin (**2**), (20*R*)-*O*-(3)-*α*-_L_-arabinopyranosyl-pregn-5-en-3*β*,20-diol (**3**), (20*R*)-*O*-(3)-*β*-_D_-glucopyranosyl(1 → 2)-*α*-_L_-arabinopyranosyl-pregn -5-en-3*β*,20-diol (**4**), canthin-6-one (**5**), cleomiscosin C (**6**), bruceolline F (**7**). In bold: significant cytotoxic effect [9, 72]. Data for Doxorubicin used as positive control are available in Table 2

### Cell cycle distribution and apoptosis

**Fig. 2:**
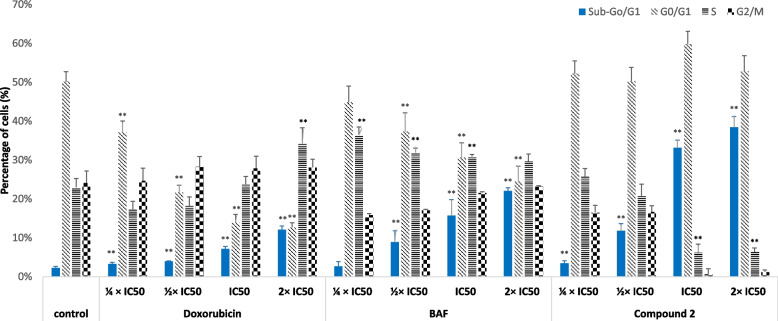
Cycle distribution of CCRF-CEM cells treated with the crude extract (BAL), hydnocarpin (**2**) from *Brucea antidysenterica*, and doxorubicin for 24 h. IC_50_ values were 17.42 µg/mL for BAL, 4.28 µM for phytochemical **2** and 0.02 µM for doxorubicin. Control cells were treated with DMSO to a final concentration of 0.1%. (**): values are significantly different to that of untreated control (*P* < 0.05)

**Fig. 3:**
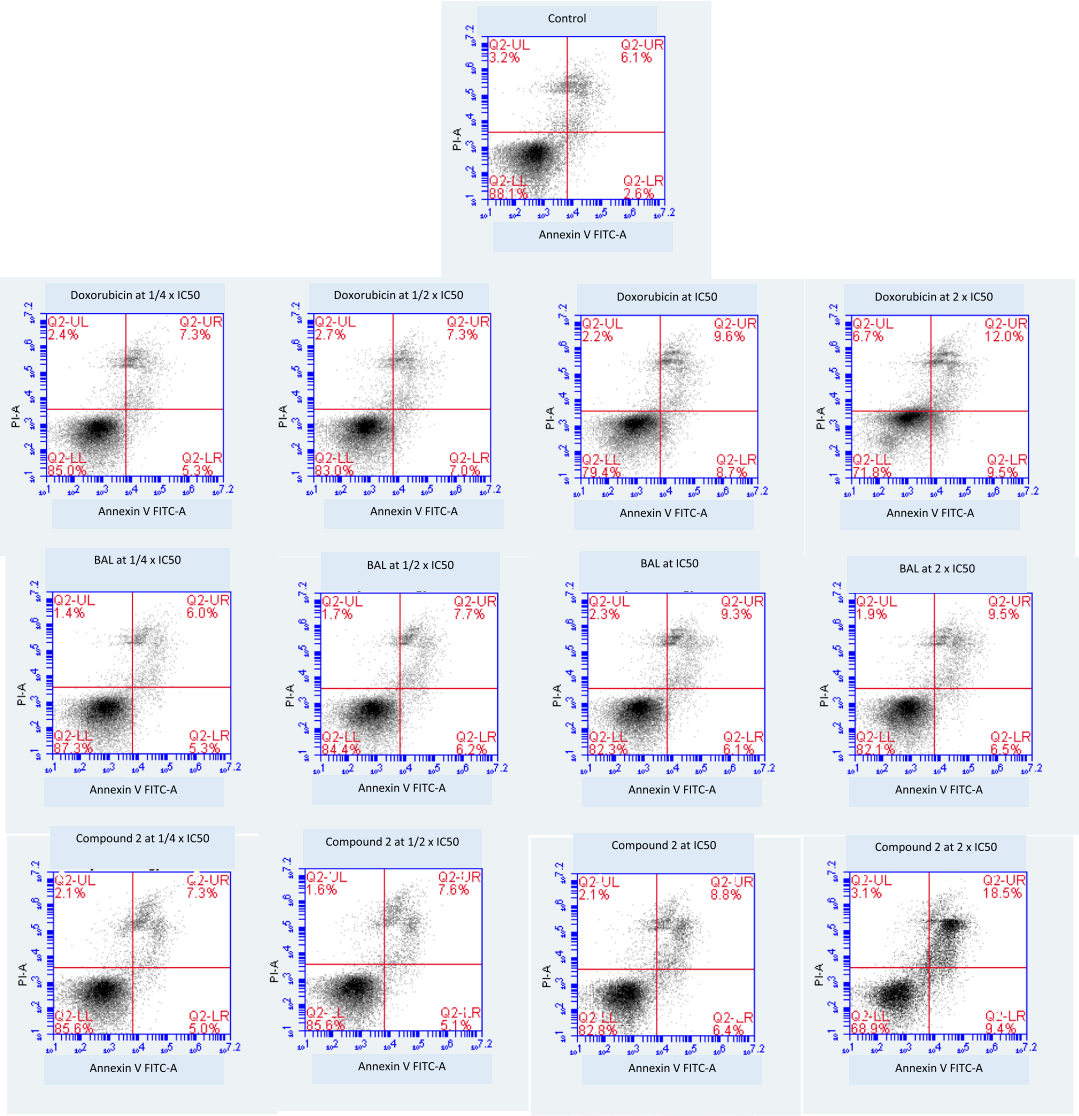
Apoptosis in CCRF-CEM cells treated with the crude extract (BAL), hydnocarpin (**2**) from *Brucea antidysenterica*, and doxorubicin for 24 h, as determined by the annexin V/PI test. Cells were measured by flow cytometric after annexin V-PI double staining. IC_50_ values were 17.42 µg/mL for BAL, 4.28 µM for phytochemical **2** and 0.02 µM for doxorubicin. Necrotic cells lose membrane integrity, allowing PI entry. Q2-LL: viable cells exhibit annexin V (-)/PI (-); Q2-LR: early apoptotic cells exhibit annexin ( +)/PI (-); Q2-UR and Q2-UL: late apoptotic cells or necrotic cells exhibit annexin V ( +)/PI ( +) or annexin V (-)/PI ( +)

The PI staining was applied to study the distribution of the various cycle phases in CCRF-CEM cells treated the test samples. The results summarized in Fig. 2 show that BAL, flavonolignan **2**, and doxorubicin induced significant changes (*p* < 0.05) of various phases of the cycle of CCRF-CEM cells in a dose-dependent manner. The cell cycle was arrested in S and G2/M phases as the result of treatment with BAL and doxorubicin, while that with compound 2 induced G0/G1 cycle arrest. The amounts of cells in sub-G0/G1 phase also change upon treatment with the test samples, with values significantly increasing (*p* < 0.05) from 2.67 ± 0.2% (1/4 × IC_50_) to 22.00 ± 1.4% (2 × IC_50_) with BAL, from 3.41 ± 0.2% (1/4 × IC_50_) to 38.40 ± 2.6% (2 × IC_50_) with flavonolignan **2**, and from 3.28 ± 1.1% (1/4 × IC_50_) to 12.05 ± 0.9% (2 × IC_50_) with doxorubicin. Apoptosis can be suggested by the increase of the cells in sub-G0/G1 phase and can be evidenced by annexin V/PI staining. For instance, the data depicted in Fig. 3 indicate that CCRF-CEM cells treated with BAL moderately underwent early apoptosis with 6.5 ± 0.4% annexin V ( +)/PI (-) cells (2 × IC_50_) *vs.* 2.6 ± 0.1% for non-treated control (Q2-UR; Fig. 3) and late apoptosis with 9.5 ± 0.76% annexin V ( +)/PI ( +) cells (2 × IC_50_) *vs.* 6.1% for non-treated control (Q2-UR; Fig. 3). Flavonolignan **2** also induced 9.4 ± 0.8% and 18.5 ± 1.3% early and late apoptotic cells at 2 × IC_50_, respectively.

### Caspase activation

**Fig. 4:**
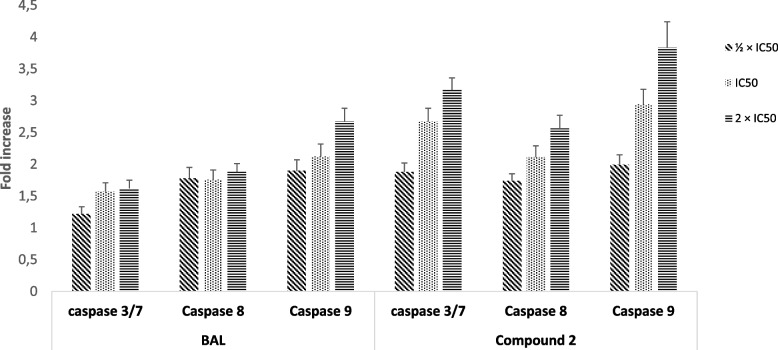
Activity of caspases in CCRF-CEM cells treated with the crude extract (BAL), hydnocarpin (**2**) from *Brucea antidysenterica* for 6 h, IC_50_ values were 17.42 µg/mL for BAL, 4.28 µM for phytochemical **2**. Caspase activity is expressed as percentage (%) compared to untreated cells. Results display mean values ± SD of three assays

The results of the caspase activity resulting in the application of BAL and flavonolignan **2** to CCRF-CEM cells are summarized in Fig. 4. At 2 × IC_50_, BAL increased the activity of caspases 3/7, 8, and 9 in CCRF-CEM cells by 1.90-fold, 2.12-fold, and 2.67-fold, respectively, whilst that with phytochemical **2** caused increased by 190-fold, 2.12-fold, and 2.67-fold. This is an indication that both the botanical (BAL) and its isolate (compound **2**) activated the caspases enzymes.

### Effects of test samples on the mitochondrial membrane and ROS production

**Fig. 5:**
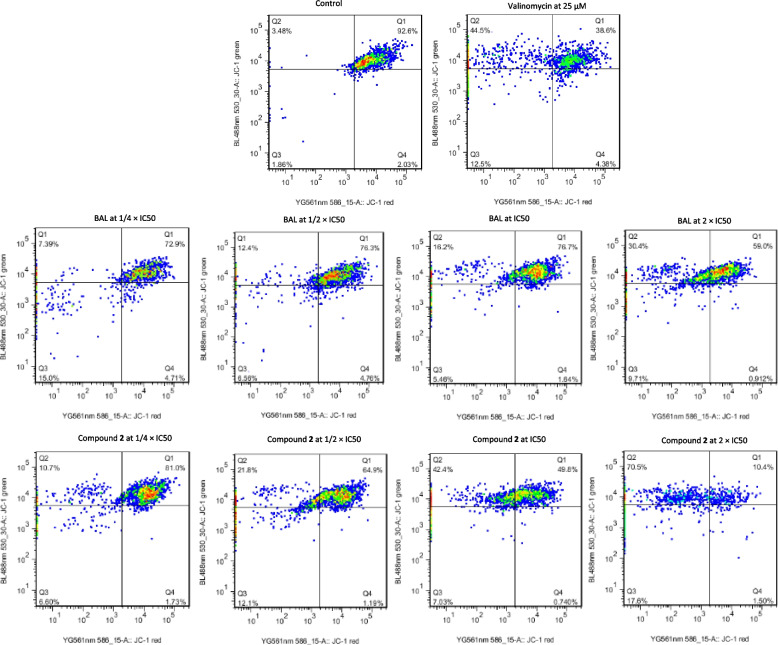
Action of the crude extract (BAL), hydnocarpin (**2**) from *Brucea antidysenterica* or valinomycin after 24 h on the mitochondrial membrane potential (MMP) of CCRF-CEM cells. IC_50_ values were 17.42 µg/mL for BAL, 4.28 µM for phytochemical **2**. Q1: cells with undamaged MMP, Q2: Cells with MMP loss, Q3-Q4: cell with disrupted MMP

After treatment of CCRF-CEM cells with BAL, flavonolignan **2**, and valinomycin, the JC-1 staining was applied to analyze the integrity of MMP. The results illustrated in Fig. 5 indicate that the application of BAL and flavonolignan **2** to CCRF-CEM cells considerably altered the MMP. The amounts of 59.0% and 10.4% intact MMP cells resulted from BAL and flavonolignan **2** applications, respectively, at 2 × IC_50_ *vs.* 92.6% for non-treated control (Q1; Fig. 5). BAL at 2 × IC_50_ also induced 30.4% cells with MMP loss (Q2; Fig. 5) and 10.6% cells with disrupted MMP (Q3 and Q4; Fig. 5) in CCRF-CEM cells whereas the phytochemical **2** caused 70.5% cells with MMP loss and 19.1% cells with disrupted MMP. The amounts of 38.8% healthy cells were noted after treatment with valinomycin (10 µM) while 44.5% cells and 16.8% had MMP loosed or disrupted, respectively.

**Fig. 6:**
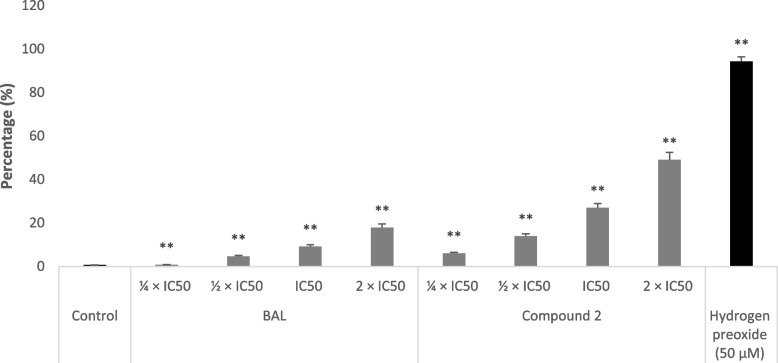
Action of the crude extract (BAL), hydnocarpin (**2**) from *Brucea antidysenterica* or hydrogen peroxide (H_2_O_2_) for 24 h on the production of reactive oxygen species in CCRF-CEM cells. IC_50_ values were 17.42 µg/mL for BAL, 4.28 µM for phytochemical **2**. Shown are mean values ± SD of three independent experiments. Control cells were treated with DMSO to a final concentration of 0.1%. (**): values are significantly different to that of untretaed control (*P* < 0.05)

Regarding ROS production, the application of BAL and flavonolignan **2** to CCRF-CEM cells induced a significant dose-dependent raise (Fig. 6). In effect, the ROS levels in treated cells increased from 0.7% (1/4 × IC_50_) to 17.80% (2 × IC_50_) and from 6.00% (1/4 × IC_50_) to 49.00% (2 × IC_50_) with BAL and phytoconstituent **12**, respectively. H_2_O_2_ (positive control) caused significant raise in the ROS levels to 94.30% at 50 µM *vs.* 0.6% in non-treated CCRF.CEM cells.

## Discussion

The importance of medicinal plants in the search of drugs is well established today [5–7]. Some illustrative anticancer molecules include vinblastine, vincristine, paclitaxel, or camptothecin [53]. Given the great diversity of plant constituents, botanicals constitute an enormous reservoir for the discovery of antiproliferative molecules to tackle the drug resistance of cancer cells. Substances intended to fight against refractory cancers must be more (*i.e.*, collateral sensitivity or hypersensitivity) or as active (normal sensitivity) on resistant than sensitive cells [48, 54]. Consequently, various resistant cell models were used in the present study to validate the role of the tested botanicals and phytochemicals to combat cancer drug resistance. Their drug-sensitive congeners were also used to determine their collateral sensitivity (hypersensitivity), *i.e*., more sensitivity of the resistant counterpart to the applied sample. They resistant model of leukemia cell line include CEM/ADR5000 cells. This cell line overexpressed P-gp (*ABCB1/MDR1*) which is the adenosine triphosphate-binding-cassette transporter (ABC). The resistant carcinoma cell lines included were MDA-MB231*/BCRP* breast cancer adenocarcinoma cells*,* HCT116 *p53*^*−/− −/−*^ colon adenocarcinoma cells*,* and U87.MG*ΔEGFR* glioblastoma multiforme cells. HCT116 *p53*^*−/−*^ cells had *p53* knockout and represent as a model of many suppressor genes [20, 25]; MDA-MB231*/BCRP* cells bear the breast cancer resistance protein (BCRP) [55]. U87.MG*ΔEGFR* cells harbor a mutation-activated *EGFR* gene (*ΔEGFR*) [56, 57]. In this work, the hypersensitivity of MDA-MB-231-*BCRP* cells *vs.* MDA-MB-231-*pcDNA* cells and U87.MGΔ*EGFR* cells *vs.* U87.MG cells to the botanical (BAL) were obtained. Hypersensitivity of CEM/ADR5000 cells *vs.* CCRF-CEM cells, HCT116 *p53*^*−/−*^ cells *vs* HCT116 *p53*^+*/*+^ cells, and U87.MGΔ*EGFR* cells *vs.* U87.MG cells to flavonolignan **2** was also achieved. These are indications that the two most active samples (BAL and compound **2**) could help to combat the cancer drug resistance. IC_50_ values lower than 20 μg/ml for plant extracts or 10 μM for phytochemicals were set as thresholds for good naturally occurring cytotoxic agents [58]. The crude methanol extract from the leaves (BAL) and stem (BAS) of *Brucea antidysenterica* (BAL) had IC_50_ ≤ 20 μg/ml on 3/9 and 1/9 cancer cell lines, respectively (Table 2). In addition, the IC_50_ of BAL was noted towards the nine cancer cell lines investigated, clearly showing that it should more likely be considered instead of BAS. Meanwhile, flavonolignan **2** had IC_50_ ≤ 10 μM on 8/9 studied cancer cell lines (Table 3). No other compounds displayed IC_50_ values lower than 10 μM suggesting that phytochemical **2** is the most active cytotoxic constituent of BAL.

The cytotoxicity of the crude extract from *Brucea antidysenterica* towards various models of drug-resistant cancer cells is reported here for the first time. Nonetheless, other cytotoxic molecules have been isolated from this taxon. The cytotoxicity of the crude extract on sensitive cancer cell lines was also reported. The root and bark extract had cytotoxic effects on A-549 lung carcinoma cells, MCF-7 breast cancer cells, and PC-3 prostate cancer cells (IC_50_ values varying from 65.1 to 80.5 μg/ml) [30]. This was also the case with the isolated compounds bruceacanthinones A (IC_50_ values = 195.5 μM on PC-3) and B (IC_50_ values = 150.3 to 160.5 μM), canthin-6-one (IC_50_ values = 170.3 to 177.3 μM), 1-methoxycanthin-6-one (IC_50_ values = 125.8 to 155.1 μM), 2-methoxycanthin-6-one (IC_50_ values = 121.5 to 130.9 μM), 2-hydroxy-1,11-dimethoxycanthin-6-one (IC_50_ values = 138.1 to 151.5 μM) and cleomiscosin A (IC_50_ values = 130.1 to 130.8 μM) [30]. Makong et al. found that cleomiscosin C was not active against the three reported cancer cell line. The low cytotoxicity of canthin-6-one and otherwise that of cleomiscosin C corroborates the results of the present study. The good cytotoxicity of hydnocarpin was reported by Bueno Pérez and his team toward 697 pre-B acute lymphoblastic leukemia cell line with the IC_50_ value of 8.7 μM [59]. The good cytotoxicity of hydnocarpin was also reported against murine L-1210 (IC_50_ values = 3.65 μg/ml), and Tmolt3 (IC_50_ values = 32.94 μg/ml) leukemia leukemia cells, KB nasopharynx cancer cells (IC_50_ values = 1.15 μg/ml), S-480 colon adenocarcinoma (IC_50_ values = 2.00 μg/ml), TE-418 osteosarcoma cells (IC_50_ values = 2.14 μg/ml), and HeLa-S3 uterine cells (IC_50_ values = 2.02 μg/ml), MB-9812 lung cancer cells (IC_50_ values = 8.18 μg/ml) [60]. Lee et al. have also reported the IC_50_ value of 20.3 μM for hydnocarpin toward SW480 colon cancer cells [61].

Apoptosis is recognized as one of the modes of cancer cell death induced by plant extracts and their consituents [9]. It was shown in this work that BAL and flavonolignan **2** induced apoptosis in CCRF-CEM cells. Apoptosis was also observed with hydnocarpin (**2**) derivatives in the lung and melanoma cancer cells via caspase activation [62]. The activation of caspases as observed in this study corroborates these previous findings. When it's stimulated, mitochondria release the pro-apoptotic proteins that activate caspases leading to apoptosis [63]. The crude extract, BAL, and hydnocarpin modified the MMP in CCRF-CEM cells and increases the levels of ROS, indicating their mode of induction of apoptosis.

## Conclusion

This work has demonstrated the antiproliferative potential of the crude extracts and the phytoconstituents of *Brucea antidysenterica* towards multifactorial drug-resistant cancer cell lines. The main cytotoxic constituents of the plant identified included hydnocarpin and 3, (3-(3-Methyl-1-oxo-2-butenyl))1*H* indole. Hydnocarpin was the most active compound and induced, together with the leave extract, the apoptotic cell death in leukemia CCRF-CEM cells. The leave extract of *Brucea antidysenterica* and hydnocarpin have good antiproliferative activities. Further in-depth studies including the investigation of more molecular targets, western blots, and in vivo anticancer studies are to be performed in way of discovering new anticancer drugs from these samples.

## Supplementary Information

Additional file 1.

## Data Availability

All data generated or analysed during this study are included in this published article and its supplementary information files.

## Abbreviations

**1**: 3, (3-(3-Methyl-1-oxo-2-butenyl))1*H* indole
**2**: Hydnocarpin
**3**: (20*R*)-*O*-(3)-*α*-_L_-arabinopyranosyl-pregn-5-en-3*β*,20-diol
**4**: (20*R*)-*O*-(3)-*β*-_D_-glucopyranosyl(1 → 2)-*α*-_L_-arabinopyranosyl-pregn -5-en-3*β*,20-diol
**5**: Canthin-6-one
**6**: Cleomiscosin C
**7**: Bruceolline F
ABC: ATP-binding cassette
BAL: Methanol extract of the leaves of *Brucea antidysenterica*
BAS: Methanol extract of the stem of *Brucea antidysenterica*
BCRP: Breast cancer resistance protein
CC: Column chromatography;
DMSO: Dimethylsulfoxide
D.R.: Degree of Resistance
EGFR: Epidermal growth factor receptor
AcOEt: Ehyl acetate
FITC: Flourescein isothiocynate
H_2_O_2_: Hydrogen peroxide
H2DCFH-DA: 2´,7´-Dichlorodihydrofluoresceine diacetate
IC_50_: 50% Inhibitory concentration
JC-1: The 5,5′,6,6′-tetrachloro-1,1′,3,3′-tetraethylbenzimidazolylcarbocyanine iodide
MDR: Multidrug resistant
MeOH: Methanol
P-gp: P-glycoprotein
PI: Propidium iodide;
RRA: Resazurin reduction assay
ROS: Reactive oxygen species
S.I.: Selectivity index
TLC: Thin layer chromatography
