# Supplementary material for: Cytotoxicity of the methanol extracts and compounds of Brucea antidysenterica (Simaroubaceae) towards multifactorial drug-resistant human cancer cell lines
Source: BMC Complement Med Ther. 2023 Feb 15;23:48. doi: 10.1186/s12906-023-03877-1 (PMC9930359; doi:10.1186/s12906-023-03877-1)
Supplement: Supplementary file 1 — Additional file 1. [file 12906_2023_3877_MOESM1_ESM.docx]

Cytotoxicity of the methanol extracts and compounds of *Brucea antidysenterica* (Simaroubaceae)towards multifactorial drug-resistant human cancer cell lines

Laetitia M. Youmbi^1,2^, Yves S. D. Makong^3^, Armelle T. Mbaveng^1,4^, Simplice B. Tankeo^1,4^, Ghislain W. Fotso^5^, Bruno L. Ndjakou^6^, Jean D. Wansi^3^, Veronique P. Beng^2^, Norbert Sewald^7^ Bonaventure T. Ngadjui^5^, Thomas Efferth^4^*, Victor Kuete^1,4^**,

*^1^Department of Biochemistry, Faculty of Science, University of Dschang, Dschang, Cameroon*

*^2^Department of Biochemistry, Faculty of Science, University of Yaoundé 1, Yaoundé, Cameroon*

***^3^****Department of Chemistry, Faculty of Science, University of Douala, Douala, Cameroon*

*^4^Department of Pharmaceutical Biology, Institute of Pharmaceutical and Biomedical Sciences, University of Mainz, Staudinger Weg 5, 55128 Mainz, Germany*

***^5^****Department of Organic Chemistry, Faculty of Science, University of Yaoundé 1, Yaoundé, Cameroon*

*^6^Department of Chemistry, Higher Teacher Training College, University of Yaoundé 1, Yaounde, Cameroon*

*^7^Organic and Bioorganic Chemistry, Faculty of Chemistry, Bielefeld University, 33501 Bielefeld, Germany.*

**Corresponding author:**

**Tel: (+49) 6131-3925751; Fax: (+49) 49-6131-3923752; E-mail:* [*efferth@uni-mainz.de*](mailto:efferth@uni-mainz.de)*; 55128 Mainz, Germany (Prof. Dr. Thomas Efferth)*

***Tel: +237 677355927; E-mail:* [*kuetevictor@yahoo.fr*](mailto:kuetevictor@yahoo.fr)*; P.O. Box 1499 Bafoussam, Cameroon (Prof. Dr. Victor Kuete)*

***Other author’s emails:***

*Laetitia M. Youmbi : laetitiaym@yahoo.com*

*Yves Salomon Makong :* *y.makong@yahoo.com*

*Armelle T. Mbaveng :* [*armbatsa@yahoo.fr*](mailto:armbatsa@yahoo.fr)

*Simplice B. Tankeo :* [*simplicetankeo@yahoo.fr*](mailto:simplicetankeo@yahoo.fr)

*Ghislain W. Fotso: ghis152001@yahoo.fr*

*Lenta Bruno : [lentabruno@yahoo.fr](mailto:lentabruno@yahoo.fr)*

*Jean Duplex Wansi : jdwansi@yahoo.fr*

*Veronique P. Beng : v.penlap@yahoo.fr*

*Norbert Sewald: norbert.sewald@uni-bielefeld.de*

*Bonaventure Tchaleu Ngadjui :* [*btngadjui@yahoo.fr*](mailto:btngadjui@yahoo.fr)

**S1. General experimental procedure**

The high-resolution ESI mass spectra and the corresponding higher collision dissociation (HCD) measurements (normalized collision energy 50%) were obtained on an Orbitrap Elite mass spectrometer (Thermo Fisher Scientific, Bremen, Germany) equipped with a HESI electrospray ion source (spray voltage 4 kV; capillary temperature 275°C, source heater temperature 40 °C; FTMS resolution 30.000). Nitrogen was used as the sheath gas. 1D and 2D NMR spectra were recorded either with a Bruker DRX 400, 500 NMR spectrometers or with an Agilent DD2 NMR (600 MHz) spectrometer. CDCl_3_, DMSO-d6 and pyridine-d5 were used as NMR solvents. Chemical shifts (δ) are quoted in parts per million (ppm) with tetramethylsilane (TMS) as the internal standard and the coupling constants (J) are given in Hz. Column chromatography were carried out on silica gel Merck 60 F254 [(0.2-0.5 mm) and (0.063-0.2mm)] 70-230 and 230-400 mesh (Darmstadt, Germany). TThin-layerchromatography plates were performed on Merck precoated silica gel 60 F254 aluminium foil. Spots were detected on TLC under UV lamp (254 and 365 nm) or by heating to 200 °C after spraying with 20% H_2_SO_4_ (v/v) solution. Different mixtures of n-hexane, EtOAc and MeOH were used as eluting solvents. Solvents were distilled prior to use.

**S2. NMR data of the isolated compounds**

Compound **1**. 3,(3-(3-methyl-1-oxo-2-butenyl))1H indole (C_13_H_13_NO) ; yellow powder soluble in MeOH,^1^H and ^13^C NMR data: EI-MS [M]^+^ peak at *m/z* 199.mp : 136-138^o^C. PubChem CID: 11805712

^1^H-NMR (MeOD 500 MHz,) *δ* (ppm): 8.33 (1H, *m*, H-4), 8.12 (1H, *s*, H-2) 7.45 (1H, m, H-7), 7.23 (1H, *dt*, *J* = 1.4,7.3 Hz, H-5), , 6.78 (1H, *dt*, *J* = 2.5,1.2 Hz, H-2’) , 2.22 (3H, *d*, *J* = 1.1 Hz, H-5’), 2.02 (3H, *d*, *J* = 1.1 Hz, H-4’). ^13^C-NMR (MeOD, 126 MHz,) *δ* (ppm): 188.7 (C-1’),152.0(C-3’), 137.1 (C-7a), 132.8 (C-2’), 125.9 (C-3a), 122.8 (C-2), 122.4 (C-5), 121.6 (C-6), 121.6 (C-4), 118.6 (C-3), 111.4 (C-7), 26.2 (C-4’), 19.5 (C-5’) [1]


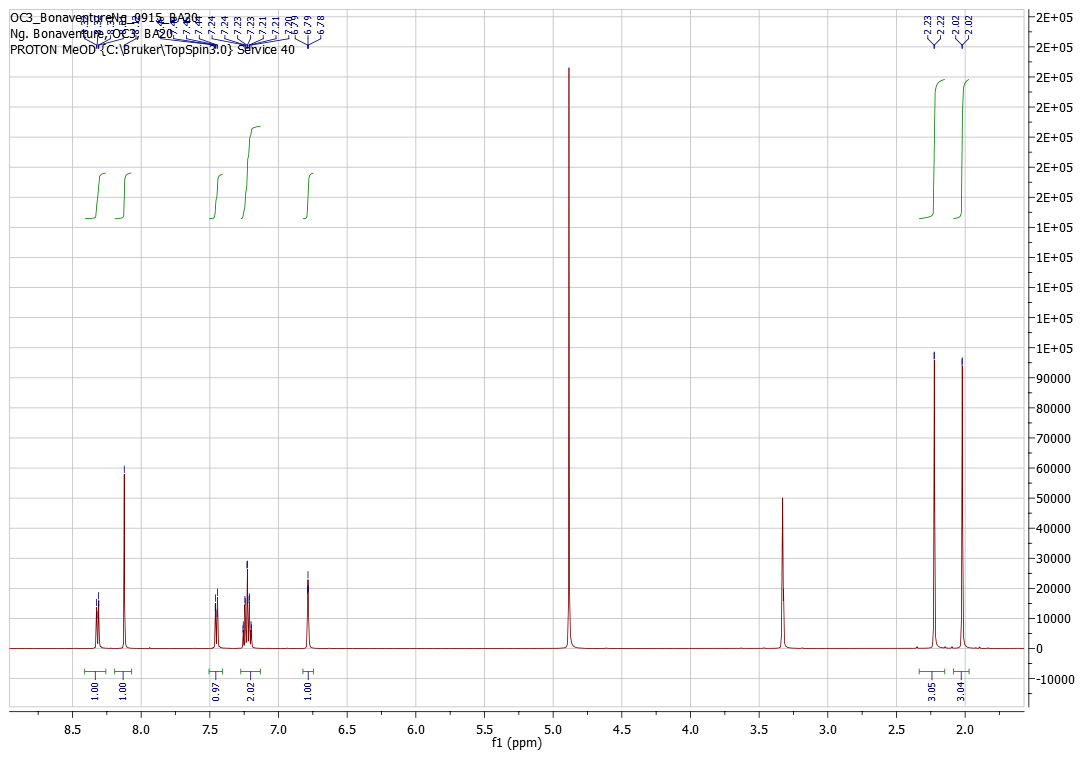


Figure S1: ^1^HNMR spectrum (500MHz, MeOD) of compound 1


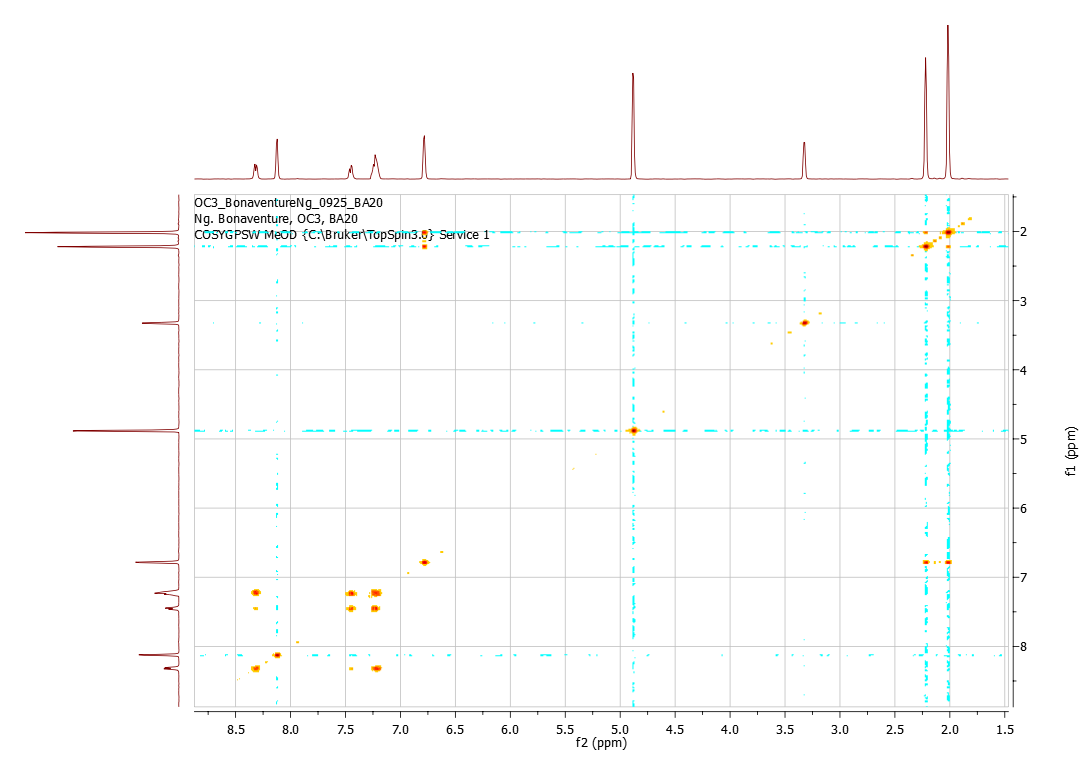


Figure S2: COSY spectrum of compound 1


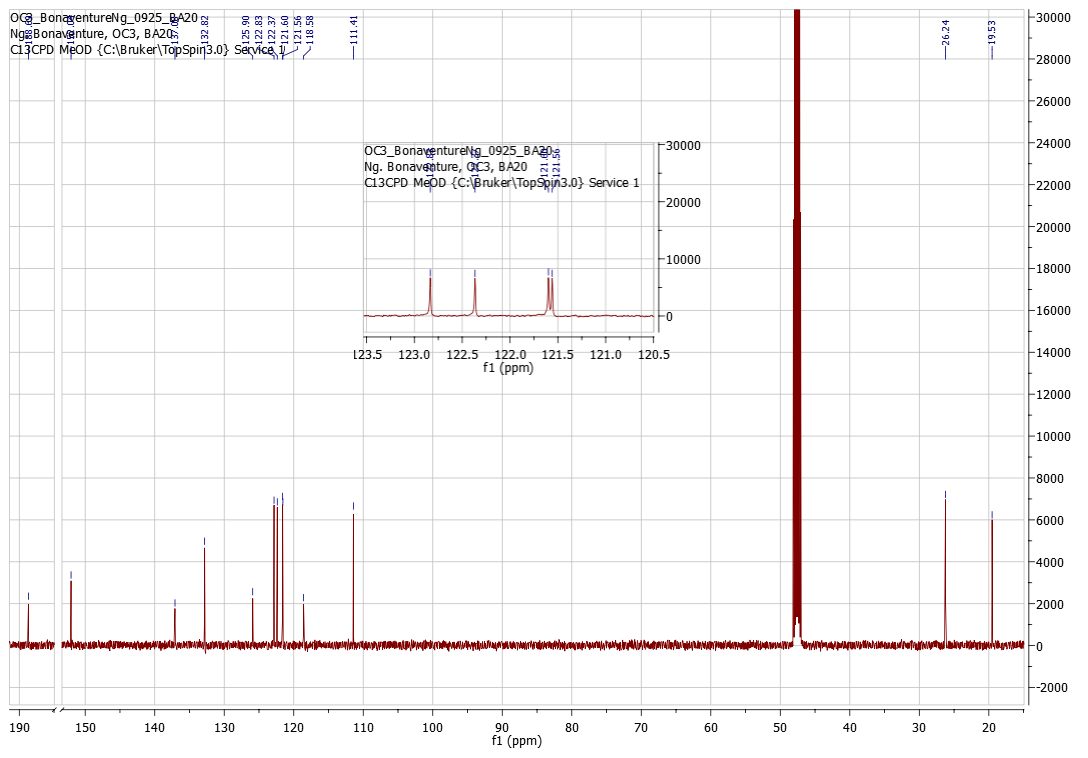


Figure S3: ^13^CNMR spectrum (126 MHz, MeOD) of compound 1


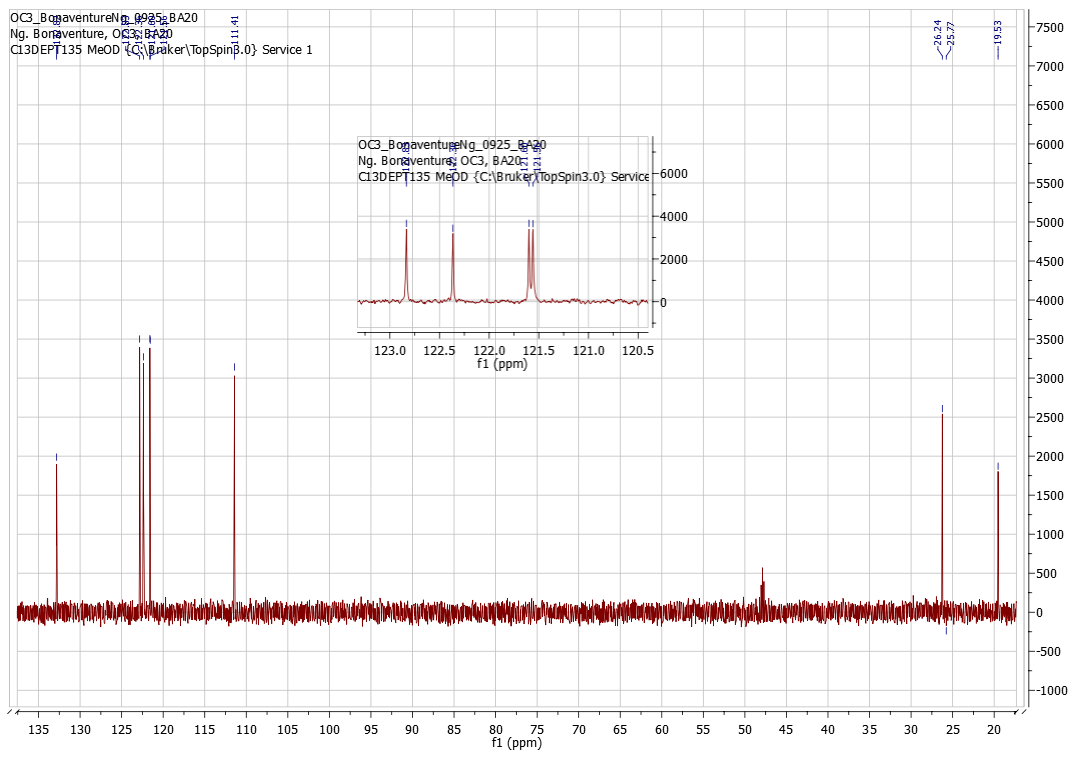


Figure S4: DEPT 135 spectrum of compound 1

Figure S5: HSQC spectrum of compound 1

Figure S6: HMBC spectrum of compound **1**

Compound **2.** Hydnocarpin (C_25_H_20_O_9_); yellow powder soluble in Pyridine-d_5,_ ^1^H and ^13^C NMR data: EI-MS [M]^+^ peak at *m/z* 464. mp : 261-263°C. PubChem CID: 5489114

^1^H NMR (Pyridine-d_5_, 600 MHz), *δ* (ppm): 13.68 (1H, s, 2.4 Hz, OH-5), 7.72 (1H, d, *J* = 2.1 Hz, H-2’), 7.53 (1H, d, *J* = 2.1 Hz, H-6’), 7.53 (1H, s, H-2’’), 7.28 (1H, d, *J* = 1.5 Hz, H-5’’), 7.26 (1H, d, *J* = 1.5 Hz, H-6’’), 6.97 (1H, s, H-3), 8.81 (1H, d, *J* = 2.0 Hz, H-8), 6.81 (1H, d, *J* = 2.0 Hz, H-6), 5.45 (1H, d, *J* = 8.1 Hz, H-7’’), 4.42 (1H, m, H-8’’), 4.19 (1H, d, *J* = 11.0 Hz, H-9b’’), 3.91 (1H, dd, *J* = 12.6, 3.6 Hz, H-9a’’), 5.45 (3H, s, CH_3_-3’). ^13^C NMR (Pyridine-d_5_, 150 MHz), *δ* (ppm): 182.5 (C-4), 165.8 (C-7), 163.5 (C-2), 162.5 (C-5), 158.3 (C-9), 148.9 (C-3’’), 148.7 (C-4’), 147.8 (C-4’’), 144.5 (C-3’), 127.6 (C-1’’), 124.6 (C-1’), 121.4 (C-6’’), 119.9 (C-6’), 117.8 (C-5’), 116.4 (C-5’’), 115.4 (C-2’), 111.8 (C-2’’), 104.9 (C-3), 104.7 (C-10), 99.9 (C-6), 94.6 (C-8), 78.6 (C-8’’), 77.4 (C-7’’), 61.2 (C-9’’), 55.7 (OCH_3_-3’) [2]


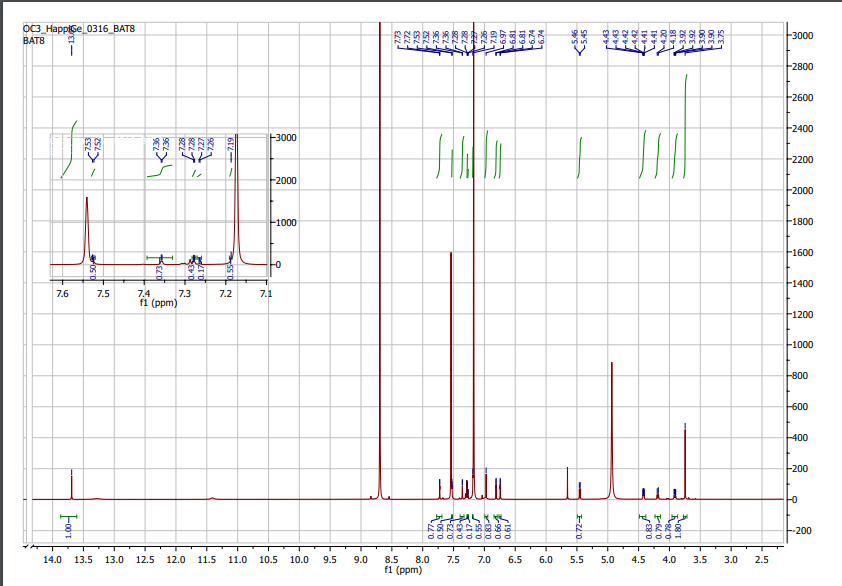


Figure S7: ^1^H NMR spectrum (600 MHz, Pyridine-d_5_) of compound 2


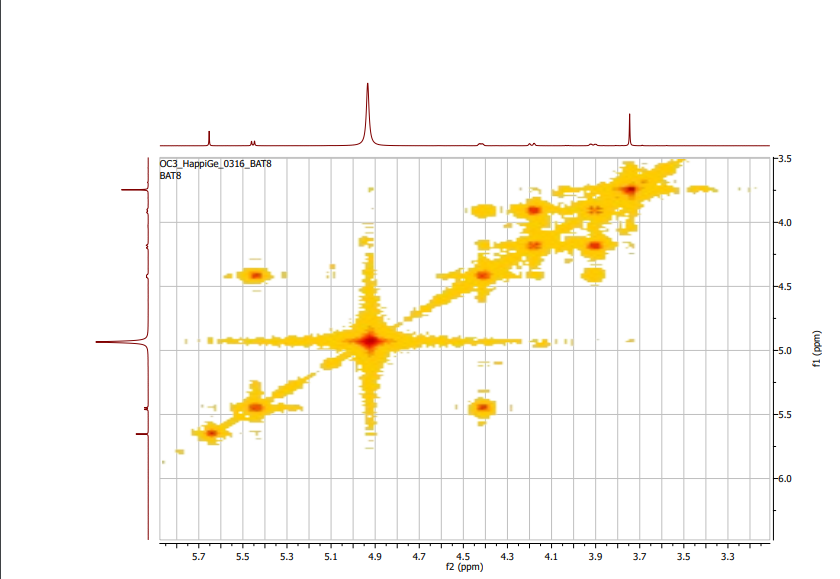


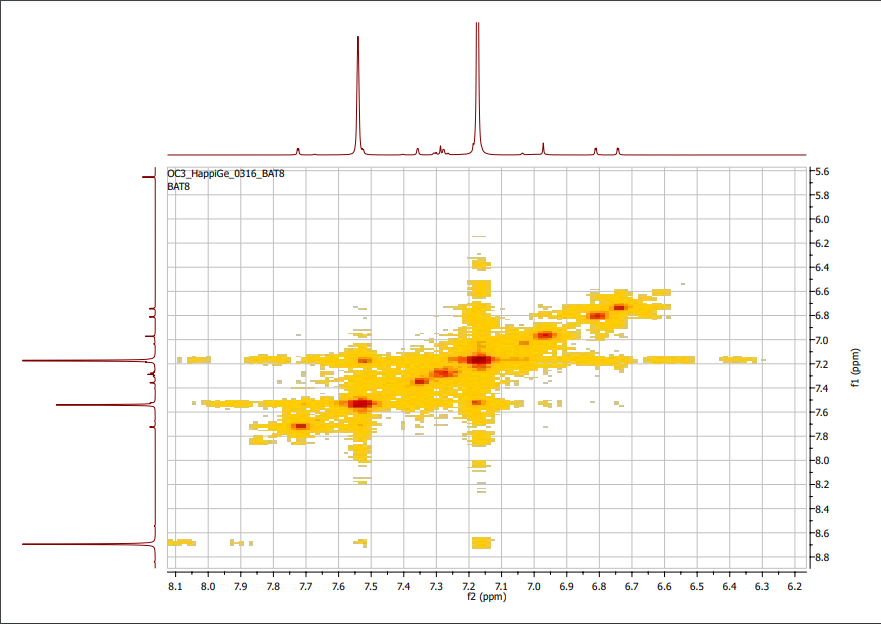


Figure S8: COSY spectrum of compound 2


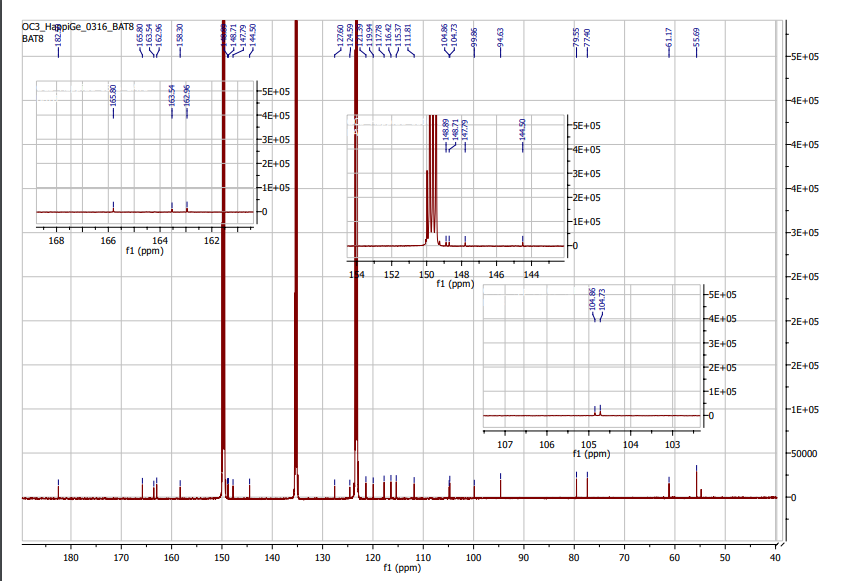


Figure S9: ^13^C NMR spectrum (150 MHz, Pyridine-d_5_) of compound 2


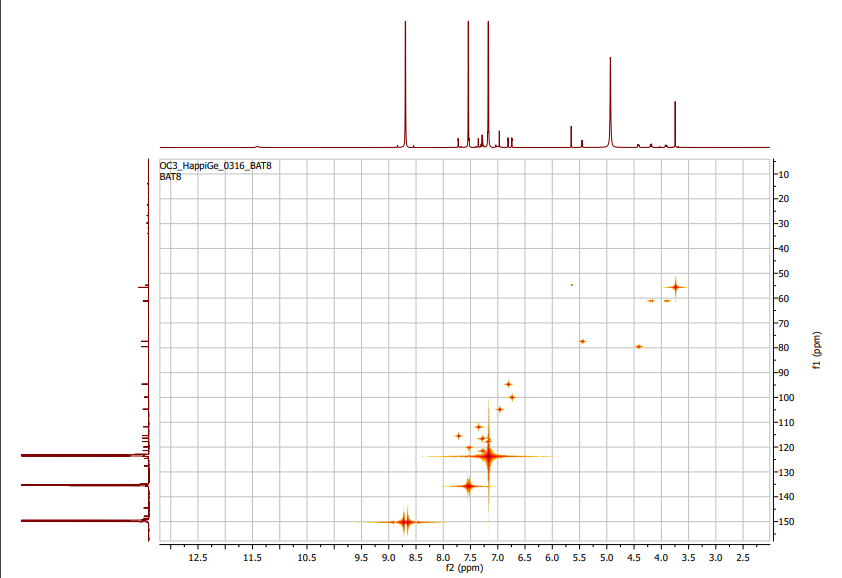


Figure S10: HSQC spectrum of compound 2


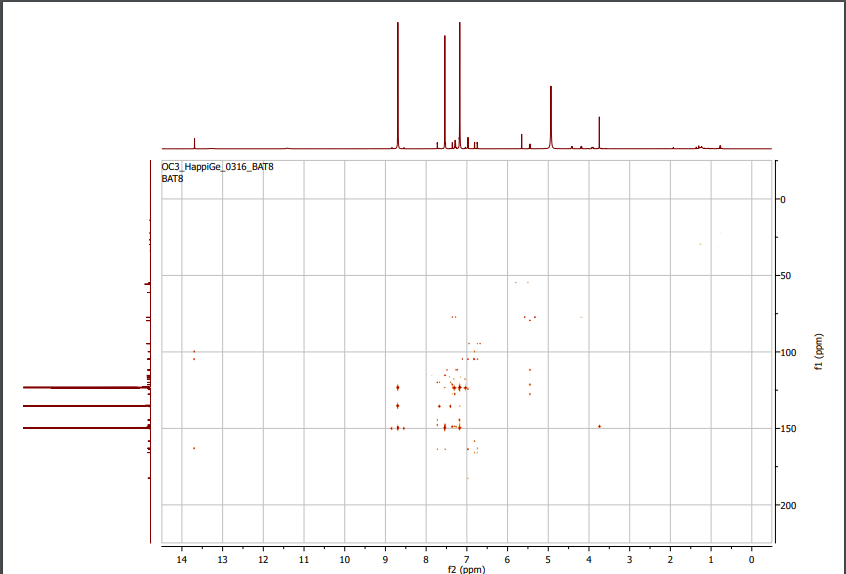


Figure S11: HMBC spectrum of compound 2

Compound 3. (20*R*)-*O*-(3)-*α*-_L_-arabinopyranosyl-pregn-5-en-3*β*,20-diol (C_24_H_42_O_6_); white powder soluble in DMSO-d_6_ ,^1^H and ^13^C NMR data: HRESIMS [M+Na]^+^ peak at m/z 473.3. mp : 258-260°C. PubChem CID: 5280794

^1^H NMR (DMSO-d_6_, 500 MHz), *δ* (ppm): 5.32 (1H, d, *J* = 13.2 Hz, H-6), 4.17 (1H, d, *J* = 6.6 Hz, H-1’), 3.64 (1H, dd, *J* = 12.1, 2.9 Hz, H-5b’), 3.59 (1H, m, H-20), 3.59 (1H, m, H-4’), 3.40 (1H, dt, *J* = 9.9, 5.9 Hz, H-3), 3.35 (1H, dd, *J* = 12.1, 2.9 Hz, H-5a’), 3.29 (1H, dd, *J* = 6.5, 4.4 Hz, H-3’), 2.28 (1H, dd, *J* = 5.3, 3.4 Hz, H-2’), 2.35 (1H, m, H-4a), 2.12 (1H, m, H-4b), 1.80 (2H, m, H-12), 1.57 (2H, m, H-16), 1.48 (2H, m, H-1), 1.46 (2H, m, H-7), 1.39 (2H, m, H-15), 1.39 (2H, m, H-11), 1.23 (1H, m, H-8), 1.23 (1H, m, H-17), 1.11 (2H, m, H-2), 1.00 (3H, d, *J* = 6.0 Hz, H-21), 0.96 (1H, m, H-14), 0.96 (3H, s, H-19), 0.88 (1H, m, H-9), 0.69 (3H, s, H-18). ^13^C NMR (DMSO-d_6_, 125 MHz), *δ* (ppm):140.9 (C-5), 121.6 (C-6), 101.9 (C-1’), 77.4 (C-3), 73.2 (C-3’), 71.1 (C-2’), 68.8 (C-20), 68.2 (C-4’), 65.8 (C-5’), 58.1 (C-17), 56.3 (C-14), 50.3 (C-9), 42.4 (C-13), 38.8 (C-4), 37.4 (C-12), 36.8 (C-10), 31.9 (C-7), 31.8 (C-8), 29.9 (C-1), 26.8 (C-15), 25.8 (C-2), 24.7 (C-16), 24.3 (C-21), 20.9 (C-11), 19.6 (C-19), 12.4 (C-18) [3]


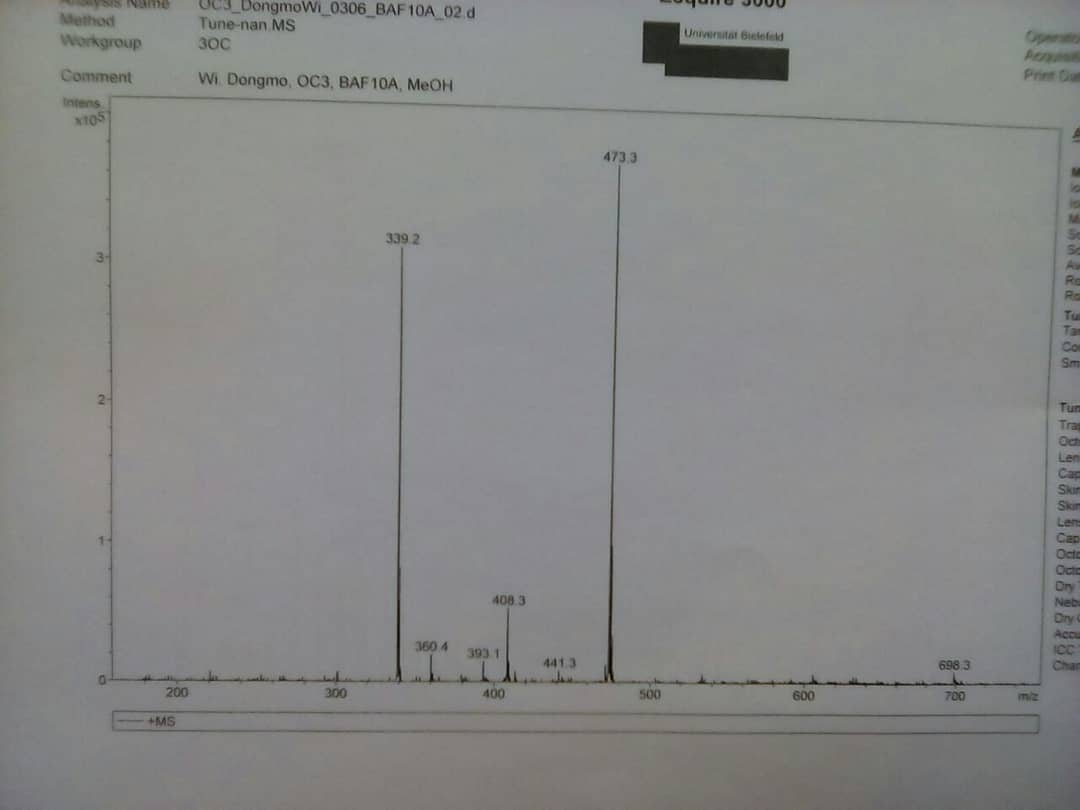


Figure S12: HRESIMS spectrum of compound 3

Figure S13: ^1^H NMR spectrum (500 MHz, DMSO-d_6_) of compound **3**

Figure S14: COSY spectrum of compound **3**

Figure S15: ^13^C NMR spectrum (125 MHz, DMSO-d_6_) of compound **3**

Figure S16: DEPT 135 spectrum of compound **3**

Figure S17: HSQC spectrum of compound **3**

 Figure S18: HMBC spectrum of compound 3

Compound 4 (20*R*)-*O*-(3)-*β*-_D_-glucopyranosyl(1→2)-*α*-_L_-arabinopyranosyl-pregn -5-en-3*β*,20-diol (C_32_H_52_O_11_) _;_ white power soluble in DMSO-d_6_,^1^H and ^13^C NMR data: ESIMS [M+Na]^+^ peak at m/z 634. mp : 258-260°C. PubChem CID: 5280794

^1^H NMR (CDCl_3_, 500 MHz), *δ* (ppm): 5.32 (1H, s, H-6), 4.52 (1H, d, *J* = 5.1 Hz, H-1’), 4.28 (1H, d, *J* = 7.8 Hz, H-1’’), 3.68 (2H, m, H-5’), 3.67 (1H, m, H-4’), 3.66 (1H, m, H-6b’’), 3.60 (1H, m, H-4’’), 3.57 (1H, m, H-2’), 3.49 (1H, m, H-20), 3.48 (1H, m, H-6a’’), 3.38 (1H, m, H-3’’), 3.22 (1H, m, H-5’’), 3.14 (1H, m, H-3’), 3.12 (1H, m, H-3), 2.97 (1H, m, H-2’’), 2.51 (2H, d, H-4), 2.39 (2H, m, H-12b), 2.16 (1H, m, H-12a), 1.96 (1H, m, H-8), 1.90 (1H, m, H-7a), 1.82 (1H, m, H-1b), 1.82 (1H, m, H-2b), 1.57 (2H, m, H-16), 1.57 (3H, d, *J* = 6.6 Hz, H-1’), 1.40 (1H, m, H-7b), 1.39 (2H, m, H-11), 1.24 (1H, m, H-17), 1.24 (1H, m, H-2a), 1.01 (1H, m, H-1a), 1.00 (2H, m, H-15), 0.98 (3H, s, H-19), 0.98 (1H, m, H-14), 0.89 (1H, m, H-9), 0.68 (3H, m, H-18). ^13^C NMR (CDCl_3_, 125 MHz), *δ* (ppm): 141.04 (C-5), 121.52 (C-6), 104.47 (C-1’’), 99.58 (C-1’), 79.95 (C-2’), 77.69 (C-3’’), 77.43 (C-3), 76.77 (C-5’’), 74.84 (C-2’’), 70.24 (C-3’), 71.17 (C-4’’), 68.76 (C-20), 66.52 (C-5’), 63.48 (C-4’), 61.36 (C-6’’), 58.11 (C-17), 56.30 (C-14), 50.27 (C-9), 42.36 (C-13), 40.00 (C-4), 38.84 (C-12), 37.33 (C-1), 36.75 (C-10), 32.00 (C-7), 31.82 (C-8), 29.87 (C-2), 25.78 (C-16), 24.80 (C-21), 24.30 (C-15), 20.90 (C-11), 19.57 (C-19), 12.41 (C-18) [4]

Figure S19: ^1^H NMR spectrum (500MHz, DMSO-d_6_) of compound **4**

Figure S20: COSY spectrum of compound **4**

Figure S21: ^13^C NMR (125MHz, DMSO-d_6_) spectrum of compound **4**

Figure S22: HSQC spectrum of compound **4**

__

Figure S23: HMBC spectrum of compound **4**

Compound **5**. Canthin-6-one(C_14_H_8_N_2_O); yellow powder soluble in MeOH,^1^H and ^13^C NMR data: EI-MS [M]^+^ peak at *m/z* 220. mp : 154-156°C. PubChem CID: 97176

^1^H-NMR (CDCl_3_/MeOD 600 MHz,) *δ* (ppm): 8.76 (1H, *d*, *J* = 5.0 Hz, H-2), 8.56 (1H, d, *J* = 8.2 Hz, H-8), 8.16 (1H, *d*, *J* = 7.7 Hz, H-11), 8.07 (1H, *d*, *J* = 5.0 Hz, H-1), 8.03 (1H, *d*, *J* = 9.8 Hz, H-4), 7.71 (1H, t, *J* = 7.8 Hz, H-9),7.55 (1H, dd, *J* = 13.3, 5.7 Hz, H-10), 6.98 (1H, d, *J* = 9.8 Hz, H-5). ^13^C-NMR (MeOD, 150 MHz,) *δ* (ppm): 159.74 (C-6),145.52 (C-2), 139.46 (C-4), 138.98 (C-13), 135.48 (C-16), 132.07 (C-15), 131.27 (C-9), 131.07 (C-14), 128.85 (C-5), 126.08 (C-10), 124.31(C-12), 123.19 (C-11), 117.00 (C-1), 116.91 (C-8)[5, 6]

Figure S24: ^1^HNMR spectrum (600MHz, MeOD) of compound 5

Figure S25: Spectre COSY spectrum of compound 5

Figure S26: ^13^CNMR spectrum (150MHz, MeOD) of compound 5

Figure S27: DEPT 135 spectrum of compound 5

Figure S28: HSQC spectrum of compound 5

Figure S29: HMBC spectrum of compound 5

### Compound **6**. Cleomiscosin C (C_21_H_20_O_9_); white power soluble in DMSO-d_6_, ^1^H and ^13^C NMR data: ESIMS [M]^+^ peak at m/z 416.mp : 254-256°C.Compound **6**. Cleomiscosin C. (C_21_H_20_O_9_); white power soluble in DMSO-d_6_, ^1^H and ^13^C NMR data: ESIMS [M]^+^ peak at m/z 416.mp : 254-256 °C.PubChem CID: 11464176

### ^1^H NMR (DMSO-d_6_, 500 MHz), *δ* (ppm): 8.59 (1H,brs, OH-4’), 7.98 (1H, d, *J* = 9.6 Hz, H-4), 6.93 (1H, s, H-5), 6.76 (1H, d, *J* = 1.2 Hz, H-2’), 6.76 (1H, d, *J* = 1.2 Hz, H-6’), 6.36 (1H, d, *J* = 9.6 Hz, H-3), 4.95 (1H, d, *J* = 7.6 Hz, H-7’), 4.40 (1H, m, H-8’), 3.80 (3H, s, CH_3_-6), 3.78 (1H, s, CH_3_-3’), 3.78 (1H, s, CH_3_-5’), 3.69 (1H, m, H-9’), 3.41 (1H, m, H-9’). ^13^C NMR (DMSO-d_6_, 125 MHz), *δ* (ppm): 160.5 (C-2), 148.5 (C-3’), 148.5 (C-5’), 145.8 (C-6), 145.3 (C-4), 138.5 (C-4’), 137.5 (C-9), 136.7 (C-7), 132.2 (C-8), 126.2 (C-1’), 113.8 (C-3), 111.8 (C-10), 106.1 (C-2’), 106.1 (C-6’), 101.3 (C-5), 78.2 (C-8’), 77.1 (C-7’), 60.3 (C-9’), 56.6 (CH_3_-6), 56.3 (CH_3_-3’), 56.3 (CH_3_-5’) [7]


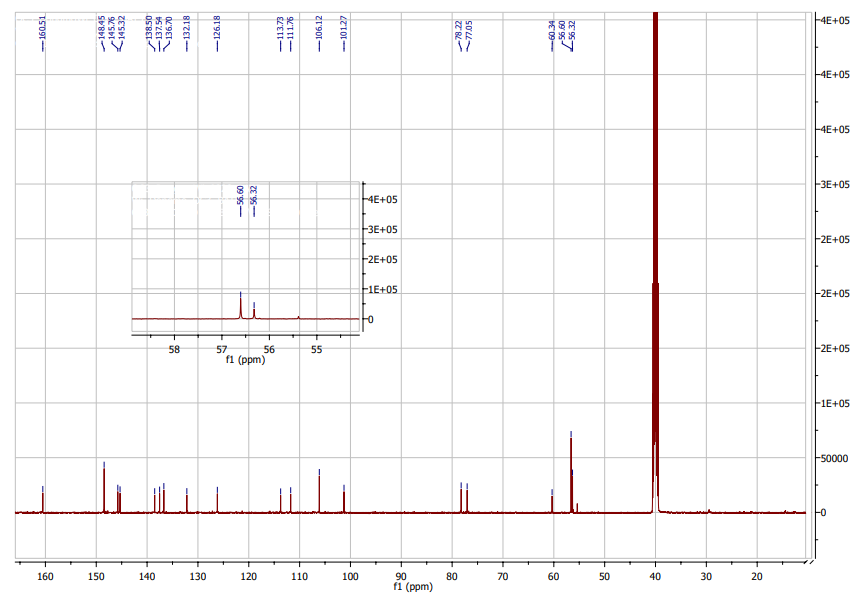


Figure S30: ^13^C NMR spectrum (125 MHz, DMSO-d_6_) of compound 6


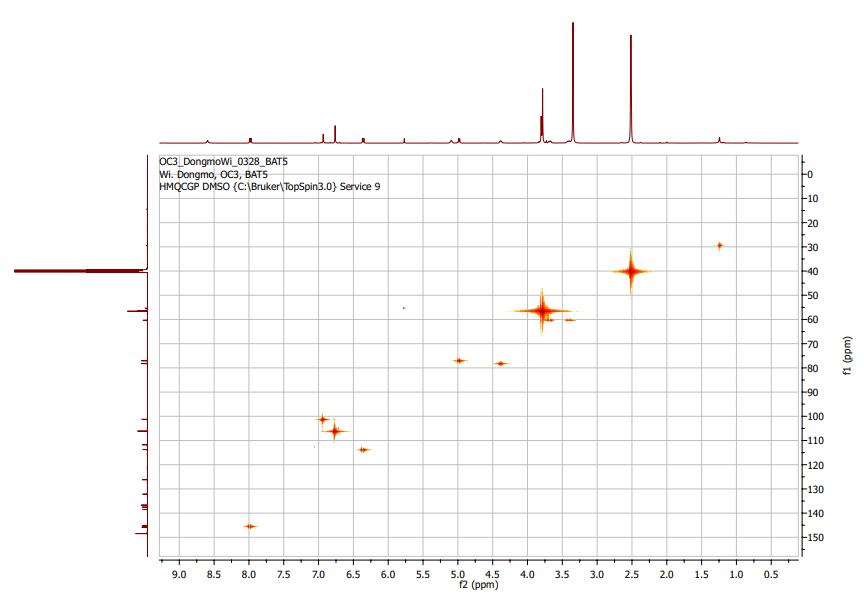


Figure S31: HSQC spectrum of compound 6


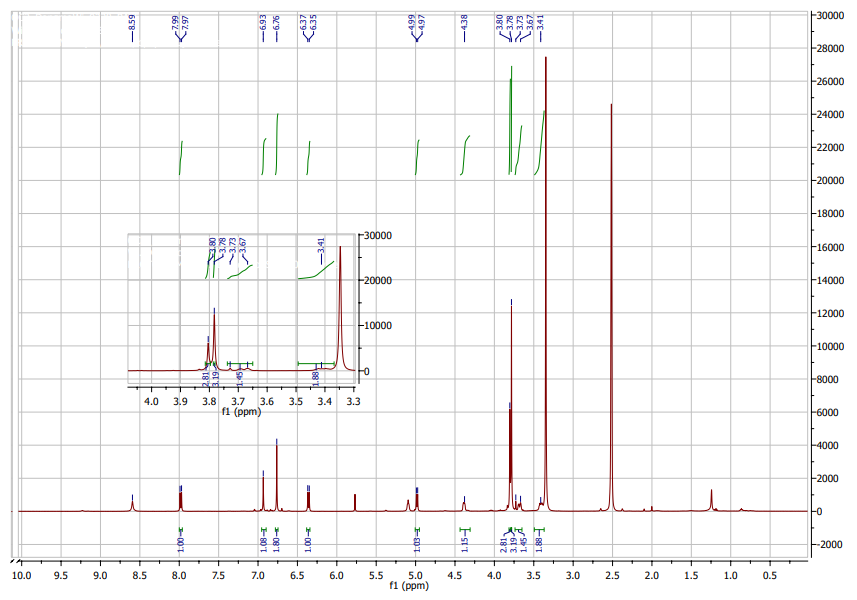


Figure S32: ^1^H NMR spectrum (500 MHz, DMSO-d_6_) of compound 6


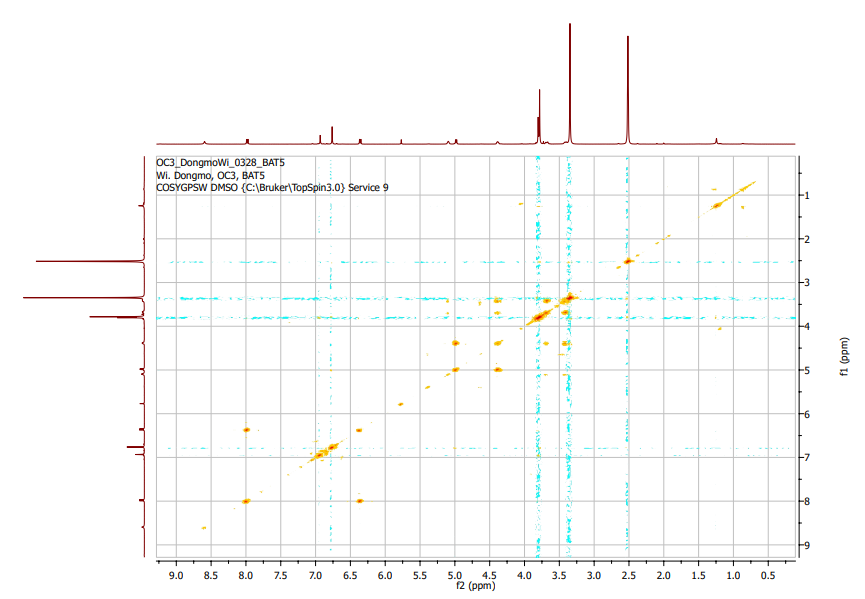


Figure S33: COSY spectrum of compound 6


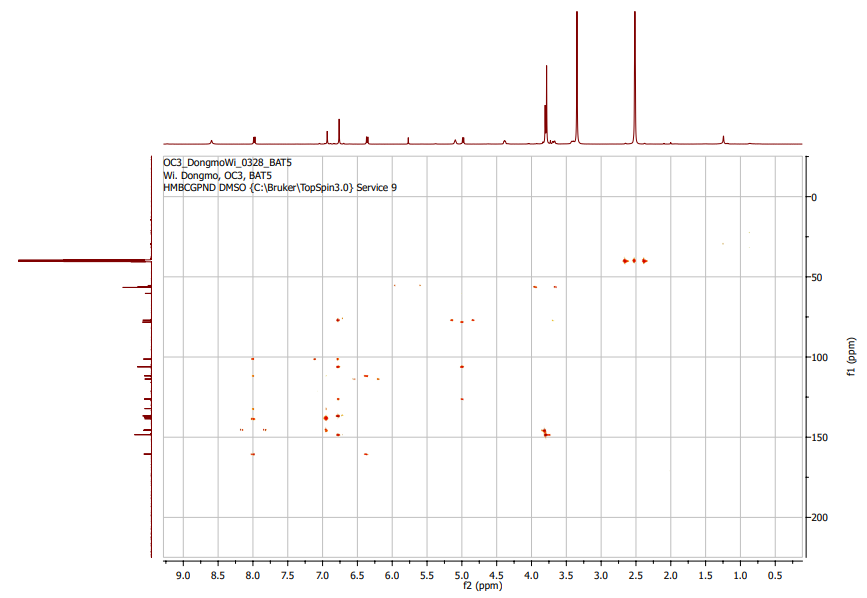


### Figure S34: HMBC spectrum of compound **6**

Compound 7. Bruceolline F or 3-(2’,3’-dihydroxy-3’-méthylbutyl)-1N-β-glucopyranosylindole (C_19_H_27_NO_7_); yellow powder soluble in DMSO-d_6_+MeOH, ^1^H and ^13^C NMR data: EI-MS [M]^+^ peak at *m/z* 381. mp : 209-211°C. PubChem CID: 38355088

^1^H NMR (500 MHz, DMSO-d_6_/MeOD), *δ* (ppm): 7.56 (1H, d, *J* = 7.8 Hz, H-4), 7.50 (1H, d, *J* = 8.2 Hz, H-7), 7.30 (1H, s, H-2), 7.14 (1H, t, *J* = 7.5 Hz, H-6), 7.05 (1H, t, *J* = 7.5 Hz, H-5), 5.39 (1H, d, *J* = 9.0 Hz, H-1’’), 3.82 (1H, m, H-2’’), 3.76 (1H, m, H-6a’’), 3.57 (1H, m, H-6b’’), 3.54 (1H, m, H-3’’), 3.51 (1H, m, H-5’’), 3.50 (1H, m, H-2’), 3.09 (1H, d, *J* = 2.0 Hz, H-1a’), 2.52 (1H, dd, *J* = 10.7, 3.0 Hz, H-1b’), 1.22 (3H, s, H-5’), 1.20 (3H, s, H-4’). ^13^C NMR (125 MHz, DMSO-d_6_/MeOD),* * (ppm): 137.2 (C-8), 128.9 (C-9), 123.9 (C-2), 121.5 (C-6), 119.3 (C-5), 119.0 (C-4), 114.1 (C-3) , 110.5 (C-7), 84.9 (C-1′’), 79.5 (C-2′), 78.1 (C-5’’), 77.8 (C-3’’), 72.4 (C-2’’), 72.3 (C-3’), 70.3 (C-4’’), 61.4 (C-6’’), 27.3 (C-1’), 26.1 (C-5’), 24.3 (C-4’) [8].

 Figure S35: Spectre ^1^H NMR (500MHz, DMSO-d6+MeOD) of compound 7

Figure S36: COSY spectrum of compound 7

Figure S37: ^13^C NMR spectrum (125 MHz, DMSO-d6+MeOD) of compound 7

Figure S38: HSQC spectrum of compound 7

Figure S39: HMBC spectrum of compound **7**

**References**

[1] Kumar V, Bulumulla HNK, Wimalasiri WR, Reisch J. Coumarins and an indole alkaloid from *Pamburus missionis*. Phytochemistry 1994, 36: 879-881.

[2] Afifi MSA, Ahmed MM, Pezzuto JM, Kinghornt A.D. Cytotoxic flavonolignans and flavones from *Verbascum sinaiticum* leaves. Phytochemistry 1993, 34: 839-841.

[3] Kamperdick C, Sung TV, Thuy TT, Tri MV, Adam G. (20R)-O-(3)-α-L-arabinopyranosyl-pregn-5-en-3β, 20-diol from *Brucea javanica*. Phytochemistry 1995; 38: 699-701.

[4] Liu JQ, Wang CF, Li XY, Chen JC, Li Y, Qiu MH. One new pregnane glycoside from the seeds of cultivated *Brucea javanica*. Arch Pharm Res 2011, 34: 1297-1300.

[5] Fukamiya N, Okano M, Aratani T, Negoro K, McPhail AT, Ju-ichi M, Lee KH. Antitumor agents, 79. Cytotoxic antileukemic alkaloids from *Brucea antidysenterica*. J Nat Prod 1986; 49, 428-434.

[6] Koike K, Ohmoto T. Quassinoids from *Quassia indica*. Phytochemistry 1994; 35, 459-463.

[7] Anil BR, Sunil KC, Sandeep K. Structures of cleomiscosins, coumarinolignoids of *Cleome viscosa* seeds. Tetrahedron 1985; 41, 209-214.

[8] Ouyang KKY, Ohmoto T. Indole alkaloids from *Brucea mollis* var. Tonkinensis. Phytochemistry 1994, 37: 575-578.
